# AIMNet2-rxn: A machine-learned potential for generalized reaction modeling on a millions-of-pathways scale

**DOI:** 10.1126/sciadv.aea1557

**Published:** 2026-10-07

**Authors:** Dylan M. Anstine, Qiyuan Zhao, Roman Zubatyuk, Shuhao Zhang, Veerupaksh Singla, Filipp Nikitin, Brett M. Savoie, Olexandr Isayev

**Affiliations:** ^1^ Department of Chemistry, Carnegie Mellon University, Pittsburgh, PA 15213, USA.; ^2^ Department of Chemical Engineering and Materials Science, Michigan State University, East Lansing, MI 48824, USA.; ^3^ Department of Chemical Engineering, Purdue University, West Lafayette, IN 47907, USA.; ^4^ Deep Principle Inc., HangZhou, Zhejiang, China.; ^5^ Department of Chemical Engineering, University of Notre Dame, Notre Dame, IN 46556, USA.

## Abstract

Mechanistic modeling of chemical transformations offers a compelling basis for understanding reactivity and allows for prediction of reaction outcomes before attempting experiments. Despite progress in machine-learned interatomic potentials (MLIPs), we demonstrate that available models lack the accuracy for diverse reaction modeling. With this motivation, we developed a general MLIP for mechanistic modeling of closed-shell carbon, hydrogen, nitrogen, and oxygen reactions, AIMNet2-rxn, using a dataset of ∼4.7 × 10^6^ range-separated density functional theory calculations. AIMNet2-rxn enables reaction modeling ∼10^6^ faster than the reference quantum mechanical (QM) methods while substantially outperforming graph-based ML, reaffirming the value using three-dimensional chemical information for training. On a test suite of well-known reaction mechanisms—such as amide formation, proton transfers, and pericyclics—AIMNet2-rxn yields 1 to 2 kilocalories per mole accuracy across reaction coordinates without retraining or system-specific fine-tuning. To exploit graphics processing unit parallelism and AIMNet2-rxn efficiency, we introduce a batched nudged elastic band procedure that readily achieves minimum energy pathway search on a millions-of-reactions scale. To demonstrate complex reaction characterization, the thermodynamics of an 11-step pathway producing hydroxymethylfurfural, the experimentally observed major product of glucose pyrolysis, is evaluated. Overall, the accuracy and efficiency afforded by AIMNet2-rxn create opportunities in high-throughput reaction discovery and deep reaction network analysis that would be infeasible with QM methods.

## INTRODUCTION

Exploiting the chemical reactivity and species selectivity of diverse reactions is foundational to nearly all chemistry applications aimed at synthesis (*1*–*4*), engineering stimuli responsiveness (*5*, *6*), or tailoring degradation (*7*, *8*), to name a few. To achieve progress in these areas, it is first necessary to establish an understanding of the chemical underpinnings that dictate the behavior of a target set of reactions so that they can be leveraged in developing novel compounds. A common strategy is to conduct a series of informative experiments that are coupled with spectroscopic and chromatographic techniques to arrive at reasonable conclusions about the mechanisms that are likely to be present and then adjust the reaction design accordingly (*9*, *10*). However, even for transformations along simplistic coordinates such as bond dissociation, it remains technically challenging to directly characterize the intricate molecular-level features displayed along a reaction pathway via experimentation (*11*). Computational mechanistic modeling with quantum mechanics (QM) calculations is the typical method to uncover such details, yielding a critical perspective on the changes in three-dimensional (3D) molecular geometry and electronic structure that govern the reaction outcomes observed experimentally. Comprehensive experimental-computational analysis is a valuable approach for collecting extensive physicochemical information needed to achieve fine-grained control of reactivity and pathway selectivity (*12*–*14*). Despite this attraction, mechanistic modeling that relies on QM calculations is both a human and computer intensive endeavor, limiting its routine use to a small number of reactions for small-to-medium sized systems. This serves as one of the main motivations for our current report, where we aim to markedly reduce the time and resources needed to perform mechanistic modeling without sacrificing accuracy using machine-learned (ML) potential energy surfaces (PESs).

A substantial focus of modern chemical discovery is the development of systems and methods that increase experimental throughput while decreasing the cost of obtaining meaningful data. For example, in 2018, Perera *et al.* (*15*) described a novel flow chemistry platform for nanomolar reaction screening and micromolar-scale synthesis, ultimately resulting in an impressive throughput of more than 1500 reactions being conducted in a 24-hour period. Matching this level of experimental data acquisition with QM-based mechanistic modeling is a nontrivial task, even for those that are the best equipped with computational resources. In the 7 years since the report described above, the emphasis on transitioning toward data-driven and high-throughput chemistry has continued to strengthen. On one hand, the embrace of ML combined with self-driving laboratories potentially offers a means of identifying novel chemical compounds absent of human intervention (*16*–*19*). High-fidelity reaction prediction is a fundamental need in this direction because automation runs the risk of wasting time and resources on unnecessary experiments that a human chemist might otherwise easily identify as likely to fail. However, as alluded to by the scale of reaction throughput mentioned above, it is too laborious a task for a human chemist to accurately predict thousands, or more, candidate reaction steps daily. On the other hand, the advent of scalable spectroscopic techniques, robotic chemistry instrumentation, and high-throughput experimental methods is a key component in the expanding capabilities of chemical discovery (*20*–*23*). The level of productivity afforded by such approaches necessitates that an ability to outline viable synthesis plans or identify operationally stable compounds must also improve. While progress is being made in selecting optimal experimental setups, for example, with Bayesian optimization (*17*) or reinforcement learning (*24*), in cases where preliminary datasets are sparse and/or the chemical design space is large, physics-based modeling can provide the chemical insight needed to plan discovery campaigns.

In this study, we introduce AIMNet2-rxn, which is an ML interatomic potential (MLIP) trained on millions of density functional theory (DFT) calculations of reactants, products, minimum energy pathways, transition states, and off-equilibrium molecular geometries. AIMNet2-rxn is the first MLIP to demonstrate broad applicability to organic chemical reactions. While other MLIPs have been reported for condensed phases under extreme conditions, system-specific reaction studies (*25*–*28*), and nonreactive molecular simulations (*29*–*35*), there has yet to be an MLIP that supports efficient and transferable mechanistic modeling. New benchmarks are reported here that demonstrate existing general-purpose MLIPs fail to describe chemical reactions. In contrast, AIMNet2-rxn is shown to efficiently perform key mechanistic modeling techniques, such as intrinsic reaction coordinate (IRC) calculations, predict reaction barriers within an agreement of 1–2 kcal mol^−1^, and identify the transition states of diverse classes of organic reactions within 0.1-Å root mean square deviation (RMSD) of DFT. Last, a batched graphics processing unit (GPU) implementation is reported that enables minimum energy pathway searches with AIMNet2-rxn to be performed for millions of reactions per day with laboratory-scale resources, opening research directions that would be otherwise unobtainable with traditional QM calculations.

## RESULTS

### Limitations of existing MLIPs

Most modern MLIP architectures are designed to represent the PES of large portions of chemical space via pretraining (*36*–*38*). Practitioners typically assert out-of-the-box applicability without retraining or fine-tuning, provided the system of interest is near the training domain. MLIPs have shown utility for simulation of equilibrium and near-equilibrium systems containing organic species; however, reports on transferability for broadly diverse reaction modeling are sparse. Prior studies for small families of reaction mechanisms suggest that training on conformers of reactive chemical systems is necessary to accurately perform mechanistic modeling tasks (*39*, *40*). We first demonstrate the limitations of existing general MLIPs for barrier height and atomic force predictions, motivating the development of reaction-focused MLIPs.

Figure 1 shows the accuracy of activation barrier inference via single-point energy calculations for the baseline AIMNet2 (A) and MACE-OFF23 (B) models on the Grambow *et al.* (*41*) dataset, which consist of chemically diverse bond rearrangements of closed-shell small-molecule organics. For completeness, we also benchmark these models on the Transition1x dataset (*42*) (fig. S1), which is chemically similar to Grambow *et al.* (*41*). These pretrained MLIP models, despite being parameterized for broad chemical space usage, result in an RMSD of 15 to 25 kcal mol^−1^ on barrier height predictions. This level of accuracy is insufficient for downstream computational reaction characterization such as kinetic analysis.

**Fig. 1.**
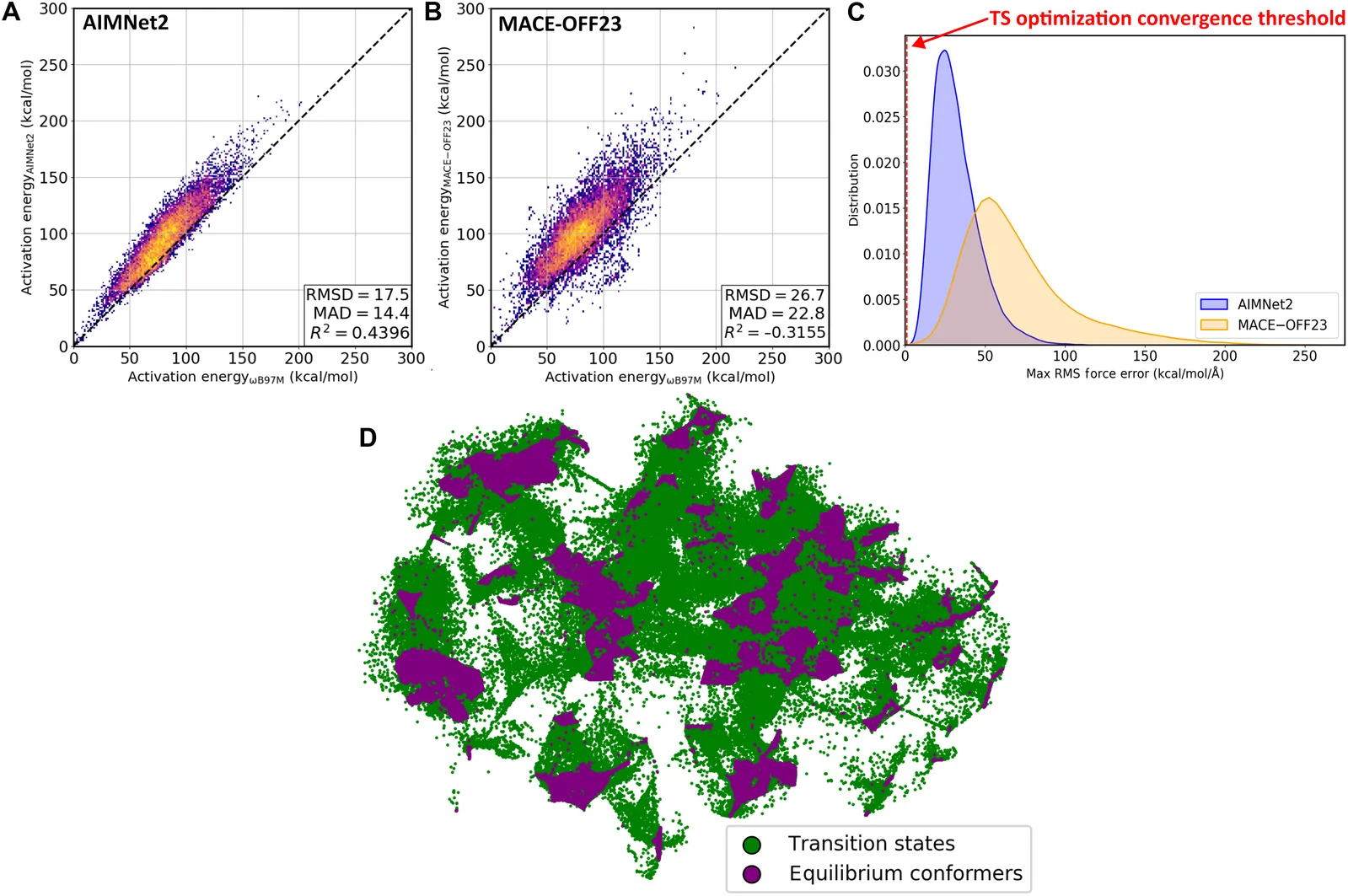
Inaccuracies of generalized ML interatomic potentials. Illustration of the failure of existing general-purpose MLIPs for describing neutral, closed-shell organic reactions, which motivates the need for reactive MLIPs. The recalculated barrier heights from the Grambow *et al.* (*41*) benchmark are shown for (**A**) AIMNet2 (*38*) and (**B**) MACE-OFF23 (*37*). (**C**) Histogram of the maximum root mean square force error for AIMNet2 and MACE-OFF23 on each of the Grambow *et al.* (*41*) transition states. (**D**) t-distributed stochastic neighbor embedding (t-SNE) plot highlighting the distinction of carbon atom local atomic environment descriptors between transition states and equilibrium (reactant and product) conformers.

Activation barrier benchmarks on provided structures do not reflect an MLIP’s ability to accurately identify reaction geometries. For exploratory in silico experiments, where structures are not known ahead of time, MLIP models can be tasked with driving PES exploration toward zeroth- (reactants and products) and first-order (transition states) critical points. Consequently, inference of atomic forces, as well as Hessians, is an additional essential performance metric to consider. In Fig. 1C, we examine the distribution of maximum force component errors of AIMNet2 (*38*) and MACE-OFF23 (*37*) for the Transition1x and Grambow *et al.* (*41*) transition states. We recomputed all the reference DFT energy and forces at the ωB97M/def2-TZVPP DFT level (*43*), which roughly matches the reference for both general MLIP models considered. There are nuances in the treatment of dispersion corrections used in each MLIP’s training data, but these differences are substantially less than the disparities observed in the atomic force predictions. Regardless, both models predict erroneously large atomic force components, indicating that they do not identify these structures as transition states. Moreover, our attempts to conduct transition state optimization using these structures with either pretrained general models resulted in a “sliding downhill” to reactant and product geometries or failures to identify negative eigenvalues, depending on the algorithm used. This inability to locate transition states prohibits these models for serving as the PES used in mechanistic modeling.

We applied t-distributed stochastic neighbor embedding (t-SNE) to provide insight into the structural differences between transition states and equilibrium geometries (Fig. 1D). The t-SNE plot of learned local environment descriptors for carbon atoms (see AIMNet2-rxn model description in Materials and Methods) delineated by transition state or reactant and product geometry in the RGD-1 dataset (*44*) is shown in Fig. 1D. Equilibrium and transition state atomic environments are observed to be distinct, suggesting that training an MLIP using only one of these classes of systems is unlikely to produce a reactive model that extrapolates to the other (or between), hence the failures of AIMNet2 and MACE-OFF23.

### Performance of AIMNet2-rxn

Here, we report a reactive MLIP, AIMNet2-rxn, with broad applicability to the mechanisms of gas phase closed-shell organic chemical transformations. AIMNet2-rxn was trained using a newly constructed dataset of 4.7 × 10^6^ ωB97M/def2-TZVPP calculations (*43*) on conformers obtained by diverse sampling strategies (Fig. 2A) such as enumerating reactant-product pairs via bond rearrangements of molecular graphs, minimum energy pathway search, and constrained molecular dynamics. As a result, conformers found in the training dataset represent equilibrium, near-equilibrium, minimum energy pathways, off-pathways geometries, and transition states, providing a basis of chemical information across the PES that is necessary to reliably identify conformers along reaction coordinates. All minimum energy pathways used to describe AIMNet2-rxn’s accuracy and transferability herein are withheld from the training set and chemically distinct external validation. The pretrained AIMNet2-rxn models are provided freely and openly (see the Data, code, and materials availability statement).

**Fig. 2.**
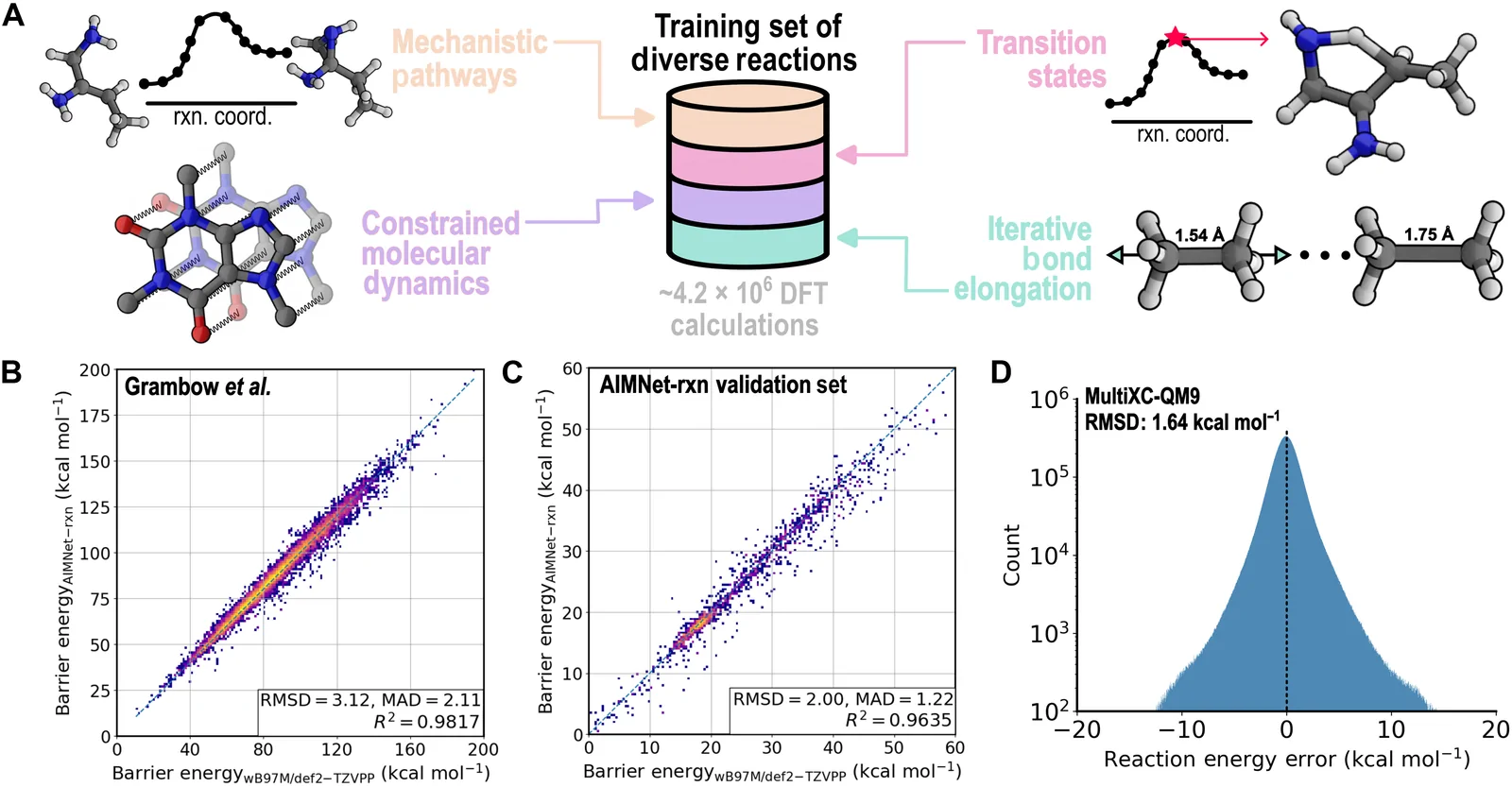
Overview of AIMNet2-rxn training and testing performance metrics. (**A**) Schematic representation of a subset of the varied sampling strategies used to construct the AIMNet2-rxn training set. Parity plots are shown for the AIMNet2-rxn barrier height predictions on (**B**) Grambow *et al.* (*41*) and (**C**) AIMNet2-rxn diverse validation sets. (**D**) Log-scale histogram of interaction energy errors on the 1.62 × 10^8^ reactant-product pairs contained in MultiXC-QM9 (*46*).

In Fig. 2B, AIMNet2-rxn barrier height predictions on the Grambow *et al.* (*41*) benchmark are reported. In comparison to the general AIMNet2 and MACE-OFF23 models shown in Fig. 1, the performance metrics have been significantly improved to 3.12 kcal mol^−1^ and 0.98 for RMSD and coefficient of determination (*R*^2^), respectively. It must be mentioned that the Grambow *et al.* (*41*) dataset is constructed by enumeration of bond rearrangements on a related small set of organic molecules. Consequently, the mechanisms composing this benchmark encompass a reduced view of chemical diversity and include several high-barrier reactions that are true pathways on the DFT PES but hold little practical value in a typical laboratory setting. These high-barrier reactions, as well as those with exotic valency and bond type changes (see figs. S2 and S3), are the largest contributors to AIMNet2-rxn mispredictions. We expand our reaction thermodynamics analysis in Fig. 2B by reporting barrier height predictions on a set of 1650 reactions consisting of numerous pericyclic reactions (Diels-Alder and [3 + 2] triazole formation), sigmatropic rearrangements, diverse mechanisms across a subset of ChEMBL compounds, and the collection of varied neutral organic closed-shell pathways reported by Hermes *et al.* (*45*). AIMNet2-rxn accurately infers barrier heights with an RMSD of 2.0 kcal mol^−1^ across this larger view of chemical reaction space exhibiting reasonable activation energies. Molecular geometries contained within this validation set are provided as part of the Supplementary Materials. In Fig. 2D, reaction energies from the 1.62 × 10^8^ reactant-product pairs (formed for 1.77 × 10^5^ geometries) in the MultiXC-QM9 test set (*46*) are reported. Consistent with barrier height inference, AIMNet2-rxn predicts these interaction energies with an RMSD of 1.64 kcal mol^−1^, indicating its value as general tool for kinetic modeling and discerning thermodynamic favorability of competing chemical pathways.

A major factor that supports the quality of AIMNet2-rxn predictions is that the model learns from the rich physicochemical information afforded by 3D molecular geometries. In contrast, previous work has pursued the development of graph-based machine learning models, operating on what is typically referred to as a 2D representation. In these approaches, molecules are defined using nodes (atoms) connected by edges (bonds), concealing geometrical information. These models predict reaction properties from chemical connectivity rather than directly modeling interaction energies. Setting aside the issue of different conformers sharing equivalent molecular graphs but distinct reaction properties, these models are found to perform worse than AIMNet2-rxn even when they are trained on more focused datasets. For reference, Chemprop (*47*), a popular graph message passing neural network used in cheminformatics research, achieves a barrier height prediction MAE of 4.05 kcal mol^−1^ on the Grambow *et al.* (*41*) dataset, nearly a factor of 2 less accurate than AIMNet2-rxn. Moreover, their model uses the same benchmark for creating the cross-validation train-test split, and, as a result, prediction accuracy severely degrades on external sets (*48*), which is a limitation also overcome by our MLIP. AIMNet2-rxn avoids this degradation in performance by learning to relate molecular geometry to interaction energy and forces, which is a process physically consistent with the reference DFT calculations, albeit at the level of neglecting explicit treatment of electronic wavefunctions.

Meaningful demonstrations of mechanistic modeling capability must include an assessment of identifying conformer geometries and energies along varied reaction coordinates. To this end, reaction pathway analysis is a collection of optimization problems where molecular geometries connecting PES minima and maxima are determined. The prediction of such chemical configurations is performed by refining initial structural estimates with atomic force minimization techniques informed by the AIMNet2-rxn PES curvature. Therefore, successfully describing chemical transitions starting only from a priori knowledge of reactants and products relates to AIMNet2-rxn’s robust transferability for both on- and off-minimum energy pathway conditions.

We report six reaction profiles obtained by the growing string method, transition state optimization, and IRC calculation with AIMNet2-rxn and DFT. These results are obtained using the same models applied in Fig. 2 without any mechanism specific training. The close comparison between AIMNet2-rxn and DFT for concerted amide bond (Fig. 3B) and β-keto ester (Fig. 3F) formations showcases that our model accurately recovers key mechanisms of diverse condensation reactions. Alkene oxidation to create an epoxide (Fig. 3D) and alcohol addition to produce a carbamate (Fig. 3G) were selected as further test cases because predicting these pathways requires AIMNet2-rxn to generalize across proton transport events, which our initial assessment identified as being challenging for lower level of theory QM approaches. AIMNet2-rxn maintains consistently high prediction accuracy for both geometry and interaction energy. In Fig. 3 (C and E), the reaction pathway analysis is extended to cycloadditions, Diels-Alder and [3 + 2] triazole synthesis, which serve as examples for our MLIP’s applicability to mechanisms where more than two bonds are rearranged. Together, these six pathways are representative of AIMNet2-rxn’s ability to model chemical reactions with minor differences in energy, typically less than 1.0 kcal mol^−1^, across entire reaction coordinates. As a general trend, we observe that the largest deviations for MLIP mechanistic modeling typically occur near the transition state. Although the reduction in prediction quality is minor, it is indicative of difficulties associated with sampling molecular geometries in highly curved regions of the PES. Addressing this challenge efficiently is a topic under ongoing investigation. Regardless, it is important to briefly comment on the composition of the AIMNet2-rxn training data to provide a broader context for the results presented in Fig. 3, particularly as it pertains to extrapolation. Our training data were constructed using chemical pathways derived by applying general bond rearrangements on bond-electron matrices, so it should be acknowledged that conformers belonging to various organic model reaction mechanisms are naturally present. As an example, in our application of a breaking-three-bonds, forming-three-bonds rule, we observe a small number of cycloadditions on model compounds, but we do not exhaustively enumerate or target mechanisms such as Diels-Alder; thus, Fig. 3 strictly shows tests of transferability.

**Fig. 3.**
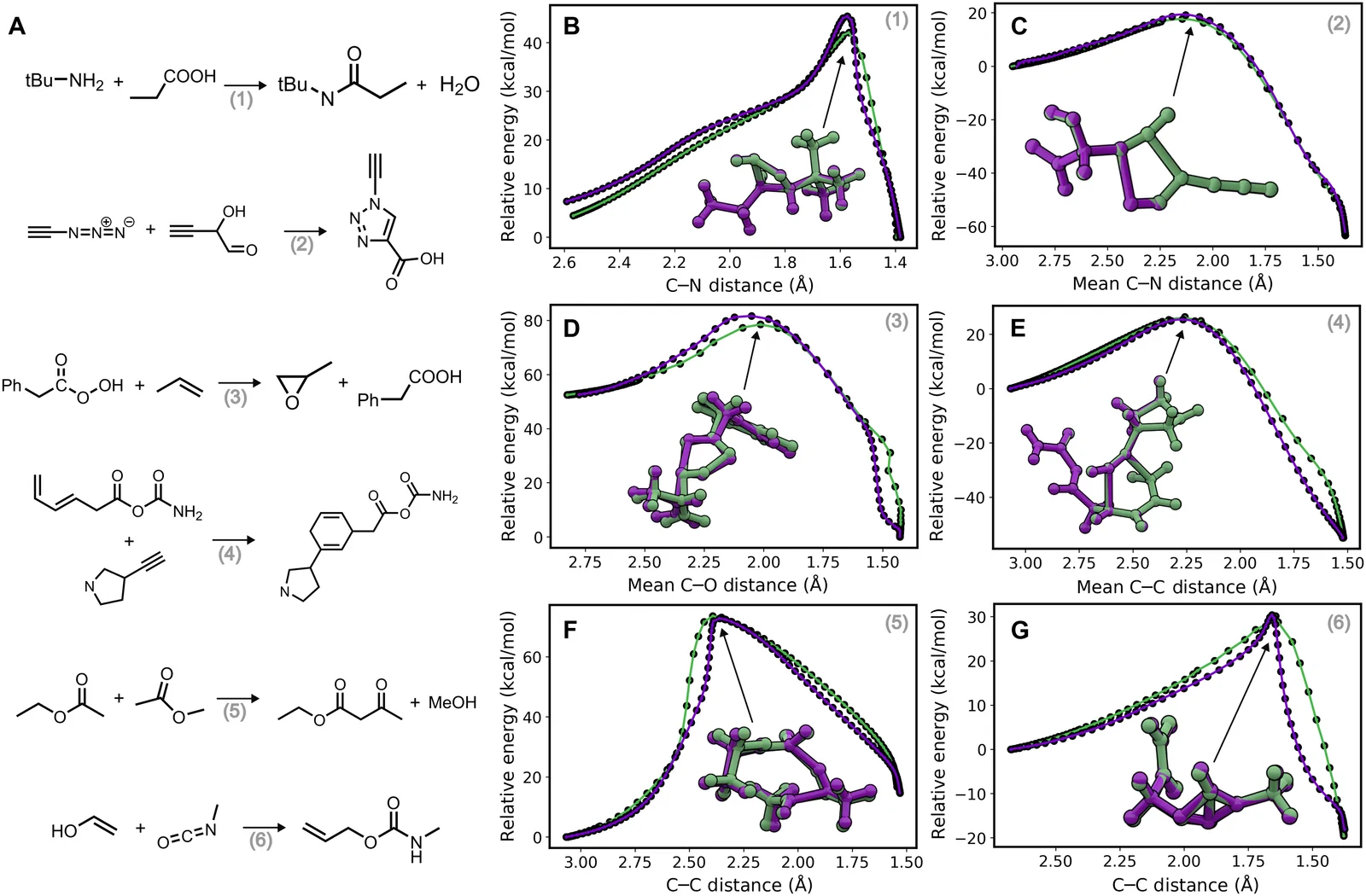
AIMNet2-rxn IRC profiles. Comparison of AIMNet2-rxn (purple) and ωB97M/def2-TZVPP (green) calculated IRCs of diverse pathways. (**A**) Overview of chemical reactions used for testing. IRC profiles and transition state structural overlaps are shown for (**B**) amide bond formation, (**C**) triazole formation, (**D**) alkene oxidation to an epoxide, (**E**) Diels-Alder cycloaddition, (**F**) β-keto ester formation, and (**G**) alcohol addition to produce a carbamate.

Figure 4A compares AIMNet2-rxn and DFT optimized transition state geometries of the validation set used in Fig. 2. For all transition state structures considered, we confirmed that each system exhibited exactly one negative frequency. Structural differences between the two methods are minor, resulting in a mean RMSD of 0.07 Å and 95% of structures being identified within a 0.17-Å threshold. This level of structural agreement is obtained approximately six orders of magnitude faster than the range-separated hybrid DFT reference optimizations, amounting to seconds of computing time per structure on a CPU. The efficiency of AIMNet2-rxn allows for frequent exact Hessian calculations, to the point that doing so on every optimization step is feasible, which leads to better convergence behavior and accurate transition state geometries (*40*, *49*). Consistently low RMSD values across these varied mechanisms and chemical compounds demonstrate that AIMNet2-rxn can reliably perform transition state optimization of general closed-shell organic chemistry reactions.

**Fig. 4.**
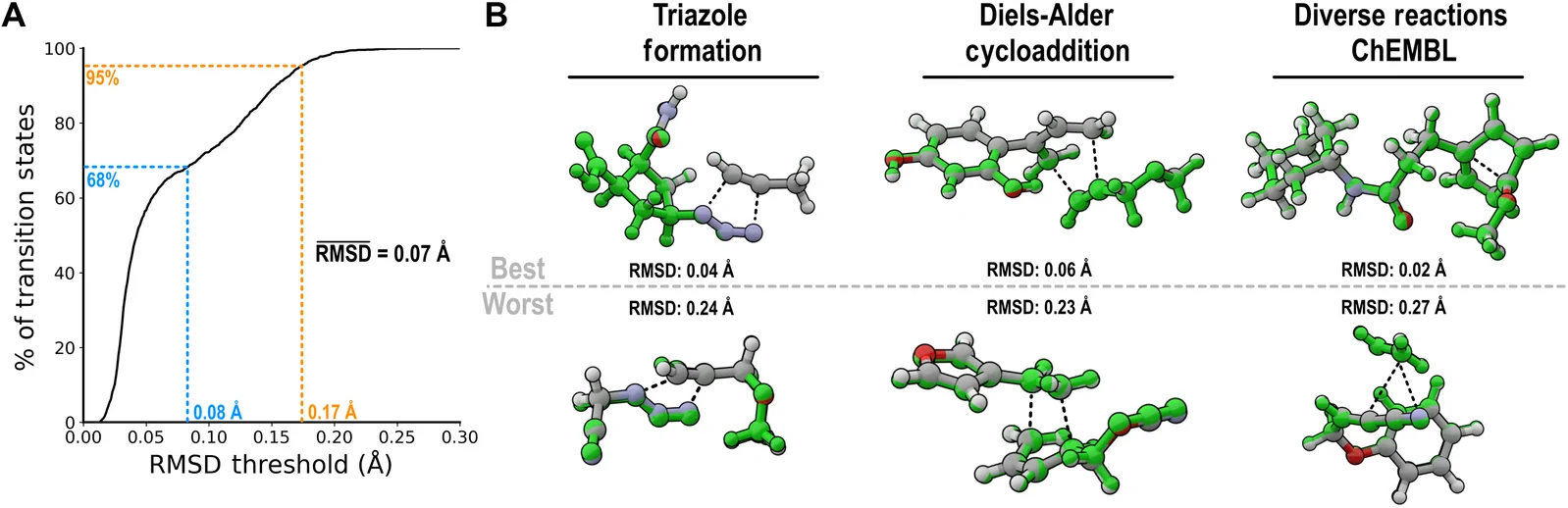
Structural agreement of AIMNet2-rxn optimized transition states. Assessment of AIMNet2-rxn (standard colors) optimized transition states compared to those obtained via ωB97M/def2-TZVPP (green) calculations on the AIMNet2-rxn validation set of 1650 reactions. (**A**) cumulative error distribution plot with optimization of thresholds of 0.08 and 0.17 Å indicating coverage of 68 and 95% of structures, respectively. (**B**) Structural overlaps for the best and worst RMSDs across validation subsets.

Structure overlaps of the best and worst AIMNet2-rxn–identified geometries across different validation subsets are shown in Fig. 4B. The observed differences are minor, even in the worst cases, and we identify that deviations are largely denominated by molecular alignment and not the reaction coordinate geometry. Therefore, we expect the quality of transition state geometries to further improve, as MLIPs mature in their treatment of intermolecular interactions (*43*). All validation and testing systems reported in Figs. 2 and 4 were manually verified to consist of reactant and product molecules that are not represented in the training set, which indicate transferability to unseen chemical reactions. In fig. S4, the accuracy of AIMNet2-rxn–predicted molecular Hessians is presented as a parity plot (*R*^2^ = 0.998) with respect to ωB97M/def2-TZVPP for a subset of reactions from the Hermes dataset.

### Batch nudged elastic band

Fully realizing the efficiency of AIMNet2-rxn for reaction characterization requires redesigning traditional methods for mechanistic modeling to allow for concurrent parallel execution. For instance, applying the nudged elastic band (NEB) method (*50*, *51*) with DFT calculations is a strategy frequently used by computational chemists to identify a minimum energy pathway that defines a chemical transformation. In this approach, a series of conformers between reactant and product geometries is interpolated and is subjected to a coupled minimization, using fictitious springs, which results in a trajectory of molecular images that approximate the reaction pathway. While NEB with QM methods have proven effective, the cost of performing numerous DFT calculations for every intermediate molecular geometry to only describe a single reaction naturally opposes high-throughput mechanistic modeling. To enable large scale reaction characterization, we report an adaptation of the NEB method that parallelizes over batches [batched NEB (BNEB)] of independent reactive systems on GPUs with AIMNet2-rxn–inferred atomic forces and energies; see Fig. 5. Here, a high-level discussion of BNEB performance is provided. However, for those interested in technical details of the implementation, a discussion of the BNEB method is provided in note S1, and the source code is available according to the Data, code, and materials availability statement.

**Fig. 5.**
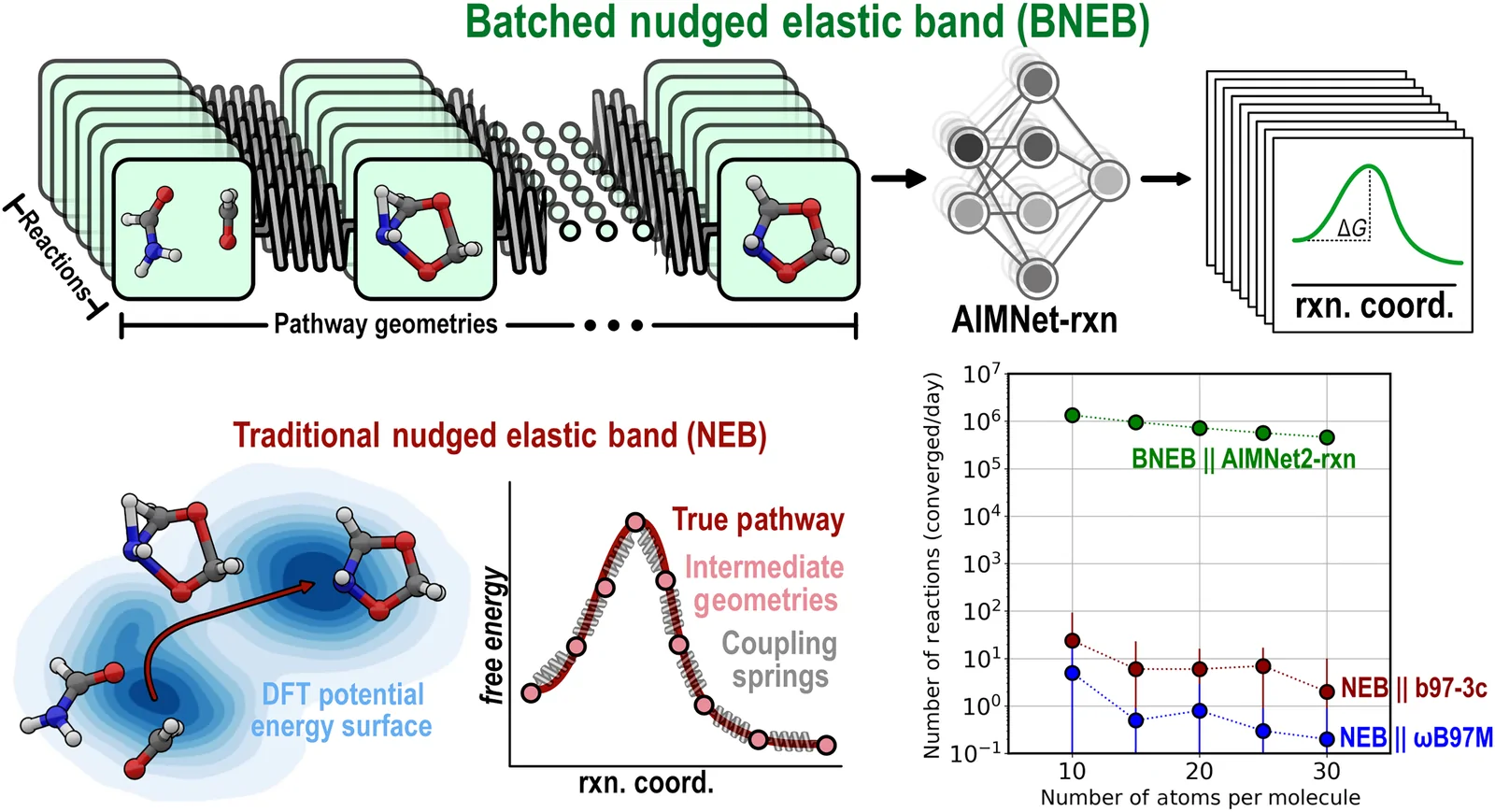
Efficiency of the BNEB strategy. Comparison of BNEB minimum energy pathway search with AIMNet2-rxn and conventional DFT NEB. Efficiency benchmarks are reported according to a throughput metric defined by the number of reactions that can be calculated daily by either BNEB/AIMNet2-rxn on a single RTX A6000 GPU or NEB/DFT parallelized over one CPU per image. See note S2 for technical details and discussion.

Figure 5 compares BNEB efficiency with NEB-DFT [b97-3c (*52*) and ωB97M/def2-TZVPP (*43*)] using a metric of reactions converged per day, where all NEB-DFT calculations are performed with a parallelization strategy of one CPU per image. For reactions containing 10 atoms, NEB-DFT yields an average throughput of 24 (b97-3c) and 5 (ωB97M/def2-TZVPP) reactions per day, whereas BNEB achieves >10^6^. Even as the size of reacting molecules grows up to 30 atoms, BNEB with AIMNet2-rxn displays an impressive reaction modeling throughput of 4.6 × 10^5^ reaction per day on a single GPU, maintaining a six orders of magnitude performance gap over the reference NEB with ωB97M/def2-TZVPP. Rigorous benchmarking between BNEB and NEB is challenging due to the underlying comparison between PES methods, variability of convergence with the reacting species and mechanisms chosen, and differences between CPU and GPU hardware. Instead, our aim is to emphasize the difference in magnitude afforded by BNEB as opposed to conducting an exact reaction characterization benchmark (see note S2). In this regard, the scale of reaction analysis that BNEB supports is unachievable for the traditional NEB-DFT approach, which is true even for impractical scenarios such as having uninterrupted and unrestricted access to available large supercomputer CPUs. As a result, BNEB with AIMNet2-rxn offers an opportunity to support previously unknown frontiers in high-throughput and complex mechanistic modeling.

While BNEB markedly improves reaction analysis throughput, it is crucial to acknowledge that high-throughput workflows that require initial pathway estimates can have many challenges to overcome for automation. Of particular importance is the sensitivity of NEB convergence to end-point conformations and the interpolant images. The impact of initial pathway generation and strategies to avoid unintended pathway convergence have been discussed, for instance, by Schmerwitz *et al.* (*53*). In the BNEB benchmarking, we carefully prepared model systems such that every pathway would converge within a typical number of NEB iterations (no more than ∼300 steps), but achieving this for parallel analysis of numerous and chemically varied mechanisms is nontrivial. For small-scale workflows, it is possible to visualize and correct pathway generation procedures using human chemical intuition. Acknowledging that such a manual practice is infeasible on a millions-of-pathways scale, we are currently developing batched versions of the sequential image-dependent pair potential (IDPP) method of Schmerwitz *et al.* (*53*) and pursing data-driven approaches for high-quality estimates of initial pathways.

### Multistep glucose reaction pathway

Understanding deep reaction networks and multistep mechanisms represents a key challenge across chemical applications (*54*, *55*). Biomass pyrolysis networks exemplify this challenge, consisting of competing reaction cascades that can yield a diversity of products (*56*). To demonstrate the practical utility of AIMNet2-rxn for addressing multimechanism problems, we examine a recently reported 11-step β-d-glucose pyrolysis pathway producing hydroxymethylfurfural (*57*). This also serves as additional validation of AIMNet2-rxn as β-d-glucose nor are any of the intermediates present in our training dataset.

The complete β-d-glucose reaction profile is displayed in Fig. 6A, with the chemistry of all intermediate molecular species provided in Fig. 6B. For every molecular geometry, single-point energies and thermal contributions to the Gibbs free energy (*G*) were calculated with ωB97M/def2-TZVPP and AIMNet2-rxn. ∆*G* comparisons between DFT and AIMNet2-rxn show an MAE and RMSD of 1.41 and 1.80 kcal mol^−1^, respectively. This good agreement supports both the quality of AIMNet2-rxn energies and the inference of accurate vibrational modes used in calculating ∆*G*. The mechanisms present in the 11-step pathway are chemically varied, including ring opening (A➔B), hydrogen transfer (B➔C), and ring formation (J➔K), to name a few. AIMNet2-rxn is found to generalize well across this diversity of chemical transformations, further establishing its use as a generalizable reactive MLIP.

**Fig. 6.**
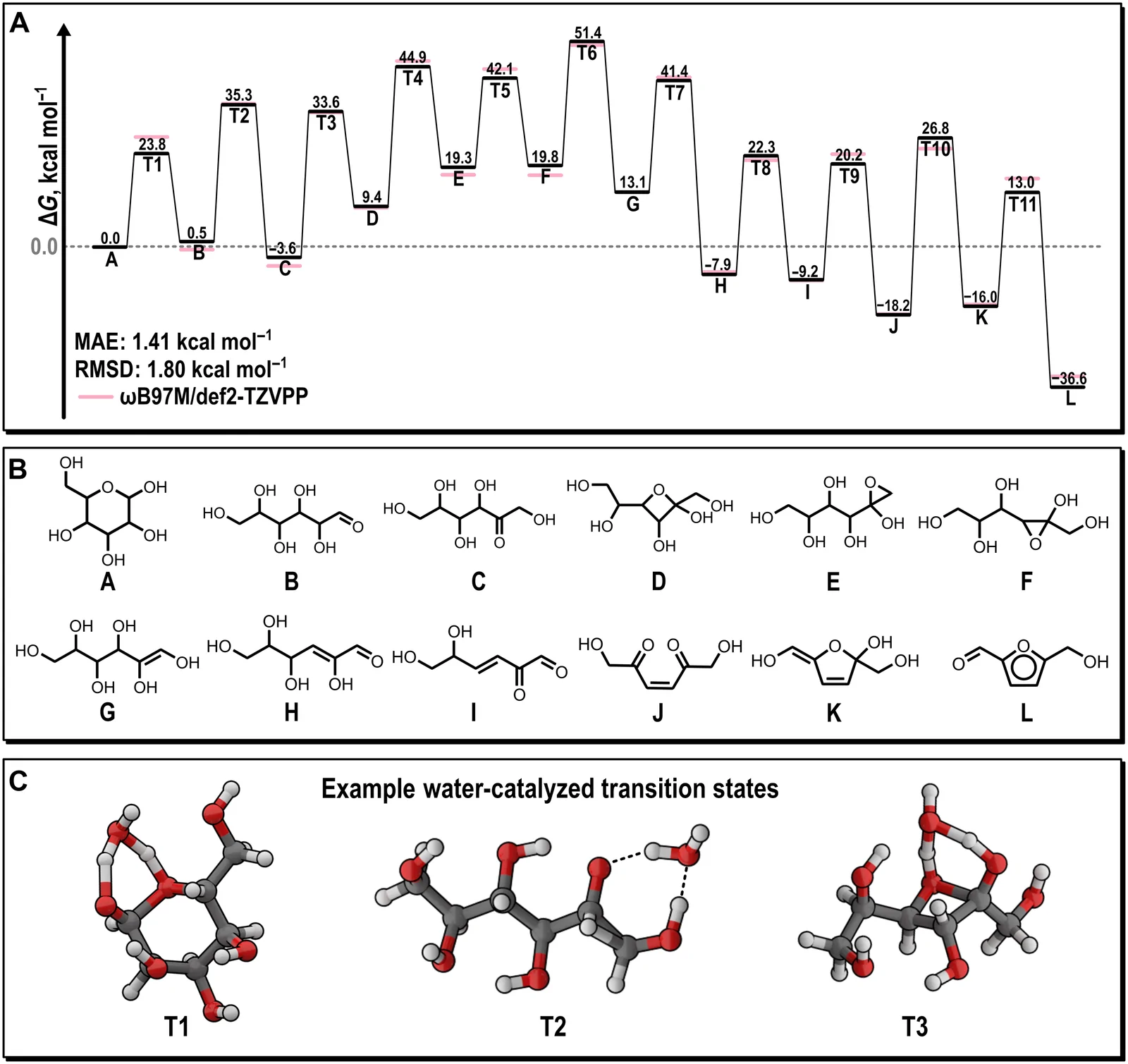
Assessment of a multistep reaction pathway with AIMNet2-rxn. Case study of a complex 11-step β-d-glucose reaction pathway that terminates at the major experimentally observed pyrolysis product. (**A**) The complete relative Gibbs free energy reaction profile characterized by AIMNet2-rxn is shown alongside accuracy metrics with respect to ωB97M/def2-TZVPP calculations. (**B**) Chemical structures for the glucose pyrolysis reactant (A), intermediates (B to K), and product (L). (**C**) Example molecular geometries of water-catalyzed transition states along the reaction profile. Free energy calculations were carried out at 298.0 K.

A physical phenomenon motivating our choice of the β-d-glucose pyrolysis pathway is the prevalence of single- and double-water–catalyzed hydrogen transfer events (Fig. 6C). We anticipated that these would be particularly challenging for AIMNet2-rxn due to being absent from the training set and the difficulty of MLIPs in learning noncovalent interactions (*58*–*60*). Considering that the energy prediction statistics are consistent with the benchmarking results presented in the previous sections, we observe that the accuracy of AIMNet2-rxn is maintained for these challenging validation cases. This demonstration should be viewed as illustrative of AIMNet2-rxn’s potential for complex pathway analysis rather than definitive validation of its accuracy across all multistep mechanisms. Extension to reaction networks involving different chemical domains, particularly those including charged intermediates or radical processes common in actual biomass conversion, will require addition of training data without architecture modification.

The computational efficiency of our MLIP model should be reiterated in the context of this case study. We were able to perform all minimum energy pathway searches, transition state optimizations, and IRC calculations in serial on CPU in a matter of minutes with AIMNet2-rxn, whereas the DFT required several days of calculation time, depending on the reaction step. The 11-step pathway considered here is a derivative of a much larger 5395-step deep reaction network determined by Woulfe and Savoie (*57*). Disregarding the generation of initial reactant and product molecular geometries, which is a separate high-throughput mechanistic modeling challenge, the entire deep reaction network can be readily generated on a laboratory desktop computer with AIMNet2-rxn. As a result, we envision that AIMNet2-rxn will play a key role in expanding the breadth and depth of deep reaction network construction and broadening the scope of mechanistic modeling problems with complex reaction spaces that can be addressed computationally.

## DISCUSSION

QM calculations have long been instrumental in uncovering the molecular-level details that give rise to experimentally observed reaction outcomes. However, their overall cost, despite the continuously growing performance in computational hardware, has limited the scale of mechanistic modeling, leaving some chemistry challenges, such as those requiring the construction of deep reaction networks (*54*, *55*), outside the purview of being routinely feasible. Current strategies, for example, YARP (*61*), seek to reduce this computational burden using stratified approaches, whereby lower-cost, lower-accuracy methods are used in downselecting candidate reactions of interest, and more expensive DFT is only used when necessary for late-stage production. In this report, we take the first steps to redesign and improve upon this paradigm with the introduction of AIMNet2-rxn. By improving upon the accuracy of low-cost methods to be near that of DFT and markedly increasing the throughput of computational characterization to a millions-of-pathways scale, we believe that AIMNet2-rxn offers transformative utility for mechanistic modeling practices. In this study, we focused on developing an MLIP for reactions of neutral organic (C, H, N, O) closed-shell pathways. In subsequent reports, AIMNet2-rxn will be extended to an expanded element set, open-shelled, and charged systems.

The scope of AIMNet2-rxn remains intentionally constrained to closed-shell organic reactions. This limitation is not merely technical but reflects a deliberate design choice to achieve high accuracy within a well-defined chemical domain rather than attempting to capture all chemical reactivity with potentially compromised performance. The marked failures we observed with existing general MLIPs on simple organic reactions emphasize the importance of this focused approach. However, this narrow scope necessarily limits the immediate applicability of our method to a subset of synthetically relevant transformations, which often involve radical mechanisms, transition metal catalysis, or heteroatoms beyond our current training set.

Nearly 70 years ago, R. Woodward wrote in a perspective that “synthesis must always be carried out by plan, and the synthetic frontier can be defined only in terms of the degree to which realistic planning is possible, utilizing all of the intellectual and physical tools available (*62*).” With the advent of high-throughput experimentation, robotic chemistry platforms, and automated discovery, it is clear that experimental synthetic capabilities are in a thriving and healthy state. As computational chemists, it is worth asking how we can best assist growing experimental productivity, or even more ambitiously, how we might reliably predict reaction outcomes before a human or robotic chemist steps foot in a laboratory. We assert that data-driven computer-aided synthesis planning (*63*) and high-throughput reaction screening offer attractive benefits among the ranks of the so-called intellectual toolkit, where AIMNet2-rxn is uniquely positioned to expand the prevalence of mechanistic modeling. A chemical challenge that is downstream to Dr. Woodward’s statement, namely, molecular or material degradation, should also be highlighted. In many cases, isolating the reaction pathways that a compound cascades through during operational failure or end of life is an outstanding challenge. Considering the success of the 11-step glucose pyrolysis pathway reported above, we envision that similar types of studies with AIMNet2-rxn will be instrumental in both previously unknown understandings of degradation and informing design decisions that lead to more robust molecules and materials.

The broader impact of this work should be viewed as establishing a foundation for reactive MLIPs rather than solving the general problem of computational reactivity prediction. Our results demonstrate that substantial improvements in both accuracy and efficiency are achievable when ML models are specifically trained for reactive systems, but considerable work remains to extend these benefits to the full spectrum of chemical reactivity. Future developments must address the inclusion of charged systems and transition metal catalysis while also confronting the fundamental challenge of incorporating environmental effects such as solvation, pressure, and temperature.

## MATERIALS AND METHODS

### Dataset construction

The molecular conformations used in constructing the AIMNet2-rxn training dataset were obtained using a diversity of sampling strategies, including constrained molecular dynamics, normal mode sampling, metadynamics with bond lengths as collective variables, iterative bond elongation, IDPP interpolations (*64*), NEB calculations, transition state optimization, and IRC calculations. In addition, a collection of equilibrium and near-equilibrium neutral organic (C, H, N, O) conformers was extracted from existing datasets, such as the AIMNet2 training set (*38*), to avoid redundant dataset generation. The entire RGD-1 dataset (*44*) was used as an initial set for providing baseline reaction coverage of the first-generation AIMNet2-rxn model. Subsequent generations of AIMNet2-rxn were produced using active learning with the well-established query-by-committee procedure using the sampling procedures outlined above with force and energy calculations from previous generation of AIMNet2-rxn. The first-generation sample set was obtained by sampling using semiempirical calculations with GFN2-xTB (*65*).

Beyond the pathways present in RGD-1, a collection of ∼400,000 additional reactions was obtained using methods similar to those in YARP (*61*) [used for RGD-1 (*44*)]. In particular, we collected out-of-sample small organic molecules (less than 40 total atoms) and applied breaking-two-bonds, forming-two-bonds and breaking-three-bonds, forming-three-bonds transformations using the bond-electron matrix of each molecular conformer. Hypothetical bond transformations were only considered if they maintained the neutral organic closed-shell chemistry of the system. This allows for both the formation of charged fragments and zwitterionic compounds so long as the net charge was 0. The updated bond matrix allowed us to assign parameters from the universal force field (*66*), which was used for minimization to the product geometry and then followed by relaxation with GFN2-xTB. These formed the end points for double-ended minimum energy pathway search with climbing image NEB with 12 images in total. Following optimization, transition states were subjected to constrained molecular dynamics calculations. We found that sampling around the transition state was crucial for obtaining the conformers necessary to ensure the quality of AIMNet2-rxn transition state optimization. Following four rounds of active learning, the final AIMNet2-rxn training set consisted of 4,685,165 geometries, which we calculated the energy, atomic forces, and molecular dipole/quadrupoles of with DFT.

### Density functional theory

All DFT calculations were carried out using the ORCA software suite (version 5.3) (*67*) with the ωB97M range-separated hybrid functional (*43*) without VV10 nonlocal correlation and the def2-TZVPP basis set. This combination was chosen on the basis of a previous analysis that reported that the ωB97 family of hybrid functionals was generally high performing on reaction modeling for their “rung” of the so-called Jacob’s ladder of QM theory (*68*). All samples were calculated to tight convergence criteria as neutral singlets in the gas phase and only contained C, H, N, or O atom types. To improve the QM calculation speed, we applied the resolution of identity for Coulomb integrals (RIJ) approximation (*69*) with the chain-of-spheres for exchange method (*70*). Regardless of their origin, all validation data reported in the results above were recalculated with a DFT protocol identical to the AIMNet2-rxn training set.

### AIMNet2-rxn architecture and training

The AIMNet2-rxn model is constructed using the recently developed AIMNet2 architecture. A detailed technical discussion of the underlying model design and operations is given in the report of Anstine *et al.* (*38*). Briefly, AIMNet2-rxn uses three message passing steps using learned representations of atomic features. In the final message passing step, each atom is assigned an information-rich 256 valued descriptor, referred to as the AIM vector. These AIM vectors are passed through a multilayer perceptron to predict atomic contributions to the total energy. Atomic forces are determined using PyTorch’s automatic differentiation (*71*) with respect to the atomic coordinates. In addition, AIMNet2-rxn derives atom-centered partial point charges during the message passing process. These are used to explicitly account for electrostatic interactions using Coulomb’s law with a short-range exponential screening, leading to AIMNet2-rxn effectively learning electrostatic contributions up to 4.6 Å implicitly. AIMNet2-rxn is trained for 2.5 × 10^6^ steps using the RAdam optimizer (*72*) and a multistep learning rate scheduler with milestones of 3 × 10^−4^, 7.5 × 10^−5^, 1 × 10^−5^, and 5 × 10^−6^. Each step amounted to sampling a random batch of 512 samples. We use the same multitarget loss functional form as was used in the base AIMNet2 model, with relative weights of 1.0, 0.2, 0.05, and 0.025 for the energy, atomic force, molecular dipole, and molecular quadrupole loss contributions, which are normalized to sum to unity. These loss weights correspond to energy and force units of electron volts and electron volts per angstrom, respectively.

### Mechanistic modeling

Mechanistic modeling methods using AIMNet2-rxn, other than BNEB, or GFN2-xTB were all carried out using the AIMNet2 plugin to pysisyphus (*73*). We extensively tested AIMNet2-rxn with pysisyphus and verified proper functionality with methods not discussed in this report such as the growing string methods. Regardless, minimum energy pathway search was conducted with the climbing image NEB method (*72*) and typically used 12 images. This number of images was adjusted when empirically deemed necessary for a small number of cases and it was only modified to include additional images. Transition state optimization was performed with the RS-P-RFO approach with a maximum trust radius of 0.05 *a*_o_. For production runs, Hessians were recalculated every step to ensure the highest quality convergence within the settings selected. During dataset construction, Hessians were calculated every five steps in the interest of calculation efficiency. IRC calculations used pysisyphus’s implementation of the EulerPC algorithm with default parameters (*13*, *74*). Convergence criteria of having a max force component of <2.5 × 10^−3^, RMS force of <3.0 × 10^−4^, max step of <1.8 × 10^−3^, and RMS step of <1.2 × 10^−3^ (in atomic units) were used for all mechanistic modeling steps. In the case of validation, e.g., comparing AIMNet2-rxn with ORCA NEB, pysisyphus settings and convergence criteria were adjusted to match the external software’s implementation and defaults.

### Batched nudged elastic band

The BNEB procedure modifies the traditional NEB algorithms to incorporate interpolation-relaxation, optimizer history and steps, geometric alignment, AIMNet2-rxn forces, and spring force calculations based on GPU kernels that operate over the leading batch dimensions. Starting from a set of aligned reactant and product geometries $R_{B,2,N,3}$, where *B* is the batch size and *N* is the number of atoms, an initial pathway ($R_{B,I,N,3}$) between end points is constructed by linear interpolation using a basis (b)

$$R_{B,I,N,3}\left(b\right)=\left(1-b\right)R_{B,0,N,3}+\left(b\right)R_{B,1,N,3},\;\text{where}\;b\in \left[0,1\right]$$ (1)

This equation converts the notation such that the subscript I refers to the number of images spanning reactants to products. For many reactions, linear interpolation is often a poor starting point for the minimum energy pathway search. To remedy this, a batched implementation of the IDPP is used to relax the geometries toward better initial structures, which has been shown to improve the efficiency of minimum energy pathway convergence (*64*). This is accomplished by first defining a set of intermediate matrices ($d^{\text{target}}$) that are obtained by linearly interpolating all atom pair distances

$$d_{B,I,N,N}^{\text{target}}\left(b\right)=\left(1-b\right)d_{B,0,N,N}+\left(b\right)d_{B,1,N,N},\;\text{where}\;b\in \left[0,1\right]$$ (2)

The potential energy of each image and atomic forces of all atoms are calculated using the difference between each image’s current internal distance matrix and the target

$${∆}_{B,I,N,N}=d_{B,I,N,N}-d_{B,I,N,N}^{\text{target}}$$ (3)

$$U_{B,I}=\sum_{i=0}^{N}\sum_{j>i}^{N}\frac{{{∆}_{B,I,i,j}}^{2}}{{d_{B,I,i,j}}^{4}}$$ (4)

$$f_{B,I,i,3}^{\text{IDPP}}=2\sum_{j=0}^{N}\frac{{∆}_{B,I,i,j}\left(1-\frac{2{∆}_{B,I,i,j}}{d_{B,I,i,j}}\right)}{{d_{B,I,i,j}}^{5}}\left(R_{B,I,i,3}-R_{B,I,j,3}\right)$$ (5)

An expanded LBFGS optimizer is applied to relax all molecular geometries with a history length (h) of the previous seven steps. During our initial testing period, it was found that a convergence criterion of $f^{\text{max}}$ < 0.01 was sufficient for high-quality minimum energy pathway estimates. Following convergence, BNEB optimization is conducted with fixed end points and the climbing image methodology. The total BNEB force acting on image is a summation of perpendicular and parallel components accounting for coupling between adjacent images

$$f_{B,I,N,3}^{\text{BNEB}}=f_{B,I,N,3}^{⊥}+f_{B,I,N,3}^{\|}$$ (6)

The perpendicular force component is calculated by projecting the AIMNet2-rxn (MLIP) force onto the perpendicular direction of the unit tangent (τ) between adjacent images

$$f_{B,I,N,3}^{⊥}=f_{B,I,N,3}^{\text{MLIP}}-\left(f_{B,I,N,3}^{\text{MLIP}}\cdot \tau_{B,I,N,3}\right)\tau_{B,I,N,3}$$ (7)

The parallel component is calculated using quadratic springs and the displacement between an image’s adjacent neighbors

$$f_{B,I,N,3}^{\|}=k\left(|R_{B,I+1,N,3}-R_{B,I,N,3}|-|R_{B,I-1,N,3}-R_{B,I,N,3}|\right)\tau_{B,I,N,3}$$ (8)

We apply a single force constant across all BNEB pathways for simplicity of implementation; however, more complex approaches based on dynamic spring weights are planned for future developments, as they can display better convergence behavior. Following a preoptimization period, the highest energy image is selected as the climbing image, and its total NEB force is instead determined by reversing the tangent direction

$$f_{B,I_{\mathrm{CI}},N,3}^{\text{BNEB}}=f_{B,I_{\mathrm{CI}},N,3}^{\text{MLIP}}-2\left(f_{B,I_{\mathrm{CI}},N,3}^{\text{MLIP},⊤}\cdot \tau_{B,I_{\mathrm{CI}},N,3}\right)\tau_{B,I_{\mathrm{CI}},N,3}$$ (9)

The climbing image index is uniquely determined for each band, which is denoted with subscript CI. To avoid so-called pathway kinks, the unit tangent is calculated according to the energy weighting scheme of adjacent images as described by Henkelman and Jónsson (*75*) with the additional batch dimension.

Similar to IDPP minimization, the BNEB optimization procedure is also carried out using the LBFGS routine (h = 7). However, the coupling between adjacent images can lead to spurious force artifacts due to misaligned geometries, and, as a result, an additional reorientation step is required. The current geometries and stored properties in the LBFGS history are aligned with the first image of each band. This is accomplished by applying batched matrix multiplication with rotation matrices ($X_{B,I,3,3}$) obtained from singular value decomposition before the typical optimizer step, for example

$$R_{B,I,N,3}^{\text{new}}=R_{B,I,N,3}X_{B,I,3,3}$$ (10)

The BNEB procedure continues until the perpendicular force components are below the defined thresholds. To improve computational efficiency, convergence is performed via dynamic masking such that matrices are shrunk in memory to only contain pathways that are currently above the force thresholds.

## Supplementary Materials

### This PDF file includes:

Figs. S1 to S6 Table S1 Notes S1 and S2

## References

[1] D. G. Brown, J. Boström, Analysis of past and present synthetic methodologies on medicinal chemistry: Where have all the new reactions gone? J. Med. Chem. 59, 4443–4458 (2016). 10.1021/acs.jmedchem.5b01409 26571338

[2] Y. Shen, J. E. Borowski, M. A. Hardy, R. Sarpong, A. G. Doyle, T. Cernak, Automation and computer-assisted planning for chemical synthesis. Nat. Rev. Methods Primers 1, 23 (2021).

[3] B. A. Wright, R. Sarpong, Molecular complexity as a driving force for the advancement of organic synthesis. Nat. Rev. Chem. 8, 776–792 (2024). 10.1038/s41570-024-00645-8 PMC11608557 39251714

[4] P. S. Baran, Natural product total synthesis: As exciting as ever and here to stay. J. Am. Chem. Soc. 140, 4751–4755 (2018). 10.1021/jacs.8b02266 29635919

[5] Q. Zhang, Y. Zhang, Y. Wan, W. Carvalho, L. Hu, M. J. Serpe, Stimuli-responsive polymers for sensing and reacting to environmental conditions. Prog. Polym. Sci. 116, 101386 (2021).

[6] D. Roy, J. N. Cambre, B. S. Sumerlin, Future perspectives and recent advances in stimuli-responsive materials. Prog. Polym. Sci. 35, 278–301 (2010).

[7] A. Samir, F. H. Ashour, A. A. A. Hakim, M. Bassyouni, Recent advances in biodegradable polymers for sustainable applications. npj Mater. Degrad. 6, 68 (2022).

[8] H. Chen, K. Wan, Y. Zhang, Y. Wang, Waste to wealth: Chemical recycling and chemical upcycling of waste plastics for a great future. ChemSusChem 14, 4123–4136 (2021). 10.1002/cssc.202100652 33998153

[9] C. J. Taylor, A. Pomberger, K. C. Felton, R. Grainger, M. Barecka, T. W. Chamberlain, R. A. Bourne, C. N. Johnson, A. A. Lapkin, A brief introduction to chemical reaction optimization. Chem. Rev. 123, 3089–3126 (2023). 10.1021/acs.chemrev.2c00798 PMC10037254 36820880

[10] P. M. Murray, F. Bellany, L. Benhamou, D.-K. Bučar, A. B. Tabor, T. D. Sheppard, The application of design of experiments (DoE) reaction optimisation and solvent selection in the development of new synthetic chemistry. Org. Biomol. Chem. 14, 2373–2384 (2016). 10.1039/c5ob01892g 26699438

[11] D. Hait, M. Head-Gordon, When is a bond broken? The polarizability perspective. Angew. Chem. Int. Ed. Engl. 62, e202312078 (2023). 10.1002/anie.202312078 37713599

[12] M. P. Maloney, B. A. Stenfors, P. Helquist, P.-O. Norrby, O. Wiest, Interplay of computation and experiment in enantioselective catalysis: Rationalization, prediction, and─correction? ACS Catal 13, 14285–14299 (2023).

[13] K. Fukui, The path of chemical reactions—The IRC approach. Acc. Chem. Res. 14, 363–368 (1981).

[14] T. Sperger, I. A. Sanhueza, F. Schoenebeck, Computation and experiment: A powerful combination to understand and predict reactivities. Acc. Chem. Res. 49, 1311–1319 (2016). 10.1021/acs.accounts.6b00068 27171796

[15] D. Perera, J. W. Tucker, S. Brahmbhatt, C. J. Helal, A. Chong, W. Farrell, P. Richardson, N. W. Sach, A platform for automated nanomole-scale reaction screening and micromole-scale synthesis in flow. Science 359, 429–434 (2018). 10.1126/science.aap9112 29371464

[16] B. Mahjour, Y. Shen, T. Cernak, Ultrahigh-throughput experimentation for information-rich chemical synthesis. Acc. Chem. Res. 54, 2337–2346 (2021). 10.1021/acs.accounts.1c00119 33891404

[17] B. J. Shields, J. Stevens, J. Li, M. Parasram, F. Damani, J. I. Martinez Alvarado, J. M. Janey, R. P. Adams, A. G. Doyle, Bayesian reaction optimization as a tool for chemical synthesis. Nature 590, 89–96 (2021). 10.1038/s41586-021-03213-y 33536653

[18] R. Pollice, G. Dos Passos Gomes, M. Aldeghi, R. J. Hickman, M. Krenn, C. Lavigne, M. Lindner-D’Addario, A. K. Nigam, C. T. Ser, Z. Yao, A. Aspuru-Guzik, Data-driven strategies for accelerated materials design. Acc. Chem. Res. 54, 849–860 (2021). 10.1021/acs.accounts.0c00785 PMC7893702 33528245

[19] S. M. Mennen, C. Alhambra, C. L. Allen, M. Barberis, S. Berritt, T. A. Brandt, A. D. Campbell, J. Castañón, A. H. Cherney, M. Christensen, D. B. Damon, J. E. de Diego, S. García-Cerrada, P. García-Losada, R. Haro, J. Janey, D. C. Leitch, L. Li, F. Liu, P. C. Lobben, D. W. C. MacMillan, J. Magano, E. McInturff, S. Monfette, R. J. Post, D. Schultz, B. J. Sitter, J. M. Stevens, I. I. Strambeanu, J. Twilton, K. Wang, M. A. Zajac, The evolution of high-throughput experimentation in pharmaceutical development and perspectives on the future. Org. Process Res. Dev. 23, 1213–1242 (2019).

[20] L. Buglioni, F. Raymenants, A. Slattery, S. D. A. Zondag, T. Noël, Technological innovations in photochemistry for organic synthesis: Flow chemistry, high-throughput experimentation, scale-up, and photoelectrochemistry. Chem. Rev. 122, 2752–2906 (2022). 10.1021/acs.chemrev.1c00332 PMC8796205 34375082

[21] T. Ha, D. Lee, Y. Kwon, M. S. Park, S. Lee, J. Jang, B. Choi, H. Jeon, J. Kim, H. Choi, H.-T. Seo, W. Choi, W. Hong, Y. J. Park, J. Jang, J. Cho, B. Kim, H. Kwon, G. Kim, W. S. Oh, J. W. Kim, J. Choi, M. Min, A. Jeon, Y. Jung, E. Kim, H. Lee, Y.-S. Choi, AI-driven robotic chemist for autonomous synthesis of organic molecules. Sci. Adv. 9, eadj0461 (2025). 10.1126/sciadv.adj0461 PMC10619927 37910607

[22] C. W. Coley, D. A. Thomas III, J. A. M. Lummiss, J. N. Jaworski, C. P. Breen, V. Schultz, T. Hart, J. S. Fishman, L. Rogers, H. Gao, R. W. Hicklin, P. P. Plehiers, J. Byington, J. S. Piotti, W. H. Green, A. J. Hart, T. F. Jamison, K. F. Jensen, A robotic platform for flow synthesis of organic compounds informed by AI planning. Science 365, eaax1566 (2019). 10.1126/science.aax1566 31395756

[23] B. Burger, P. M. Maffettone, V. V. Gusev, C. M. Aitchison, Y. Bai, X. Wang, X. Li, B. M. Alston, B. Li, R. Clowes, N. Rankin, B. Harris, R. S. Sprick, A. I. Cooper, A mobile robotic chemist. Nature 583, 237–241 (2020). 10.1038/s41586-020-2442-2 32641813

[24] J. L. Rapp, D. M. Anstine, F. Gusev, F. Nikitin, K. H. Yun, M. A. Borden, V. Bhat, O. Isayev, F. A. Leibfarth, Design of tough 3D printable elastomers with human-in-the-loop reinforcement learning. chemrxiv-2025-w1563 [Preprint] (2025). 10.26434/chemrxiv-2025-w1563. 40653448

[25] R. K. Lindsey, S. Bastea, S. Hamel, Y. Lyu, N. Goldman, V. Lordi, ChIMES Carbon 2.0: A transferable machine-learned interatomic model harnessing multifidelity training data. npj Comput. Mater. 11, 26 (2025).

[26] S. Zhang, M. Z. Makoś, R. B. Jadrich, E. Kraka, K. Barros, B. T. Nebgen, S. Tretiak, O. Isayev, N. Lubbers, R. A. Messerly, J. S. Smith, Exploring the frontiers of condensed-phase chemistry with a general reactive machine learning potential. Nat. Chem. 16, 727–734 (2024). 10.1038/s41557-023-01427-3 PMC11087274 38454071

[27] X. Guan, J. P. Heindel, T. Ko, C. Yang, T. Head-Gordon, Using machine learning to go beyond potential energy surface benchmarking for chemical reactivity. Nat. Comput. Sci. 3, 965–974 (2023). 10.1038/s43588-023-00549-5 38177593

[28] Z. Benayad, R. David, G. Stirnemann, Prebiotic chemical reactivity in solution with quantum accuracy and microsecond sampling using neural network potentials. Proc. Natl. Acad. Sci. U.S.A. 121, e2322040121 (2024). 10.1073/pnas.2322040121 PMC11161780 38809704

[29] J. Gasteiger, F. Becker, S. Günnemann, GemNet: Universal directional graph neural networks for molecules. arXiv:2106.08903 [physics.comp-ph] (2024).

[30] A. Bochkarev, Y. Lysogorskiy, R. Drautz, Graph atomic cluster expansion for semilocal interactions beyond equivariant message passing. Phys. Rev. × 14, 021036 (2024).

[31] G. Simeon, G. Fabritiis, TensorNet: Cartesian tensor representations for efficient learning of molecular potentials. arXiv:2306.06482 [cs.LG] (2023).

[32] A. Kabylda, J. T. Frank, S. Suárez-Dou, A. Khabibrakhmanov, L. M. Sandonas, O. T. Unke, S. Chmiela, K.-R. Müller, A. Tkatchenko, Molecular simulations with a pretrained neural network and universal pairwise force fields. J. Am. Chem. Soc. 147, 33723–33734 (2025). 10.1021/jacs.5c09558 PMC12447504 40886167

[33] S. Batzner, A. Musaelian, L. Sun, M. Geiger, J. P. Mailoa, M. Kornbluth, N. Molinari, T. E. Smidt, B. Kozinsky, E(3)-equivariant graph neural networks for data-efficient and accurate interatomic potentials. Nat. Commun. 13, 2453 (2022). 10.1038/s41467-022-29939-5 PMC9068614 35508450

[34] A. Musaelian, S. Batzner, A. Johansson, L. Sun, C. J. Owen, M. Kornbluth, B. Kozinsky, Learning local equivariant representations for large-scale atomistic dynamics. Nat. Commun. 14, 579 (2023). 10.1038/s41467-023-36329-y PMC9898554 36737620

[35] J. Zeng, D. Zhang, D. Lu, P. Mo, Z. Li, Y. Chen, M. Rynik, L. Huang, Z. Li, S. Shi, Y. Wang, H. Ye, P. Tuo, J. Yang, Y. Ding, Y. Li, D. Tisi, Q. Zeng, H. Bao, Y. Xia, J. Huang, K. Muraoka, Y. Wang, J. Chang, F. Yuan, S. L. Bore, C. Cai, Y. Lin, B. Wang, J. Xu, J.-X. Zhu, C. Luo, Y. Zhang, R. E. A. Goodall, W. Liang, A. K. Singh, S. Yao, J. Zhang, R. Wentzcovitch, J. Han, J. Liu, W. Jia, D. M. York, E. Weinan, R. Car, L. Zhang, H. Wang, DeePMD-kit v2: A software package for deep potential models. J. Chem. Phys. 159, 054801 (2023). 10.1063/5.0155600 PMC10445636 37526163

[36] S. Takamoto, C. Shinagawa, D. Motoki, K. Nakago, W. Li, I. Kurata, T. Watanabe, Y. Yayama, H. Iriguchi, Y. Asano, T. Onodera, T. Ishii, T. Kudo, H. Ono, R. Sawada, R. Ishitani, M. Ong, T. Yamaguchi, T. Kataoka, A. Hayashi, N. Charoenphakdee, T. Ibuka, Towards universal neural network potential for material discovery applicable to arbitrary combination of 45 elements. Nat. Commun. 13, 2991 (2022). 10.1038/s41467-022-30687-9 PMC9151783 35637178

[37] D. P. Kovács, J. H. Moore, N. J. Browning, I. Batatia, J. T. Horton, Y. Pu, V. Kapil, W. C. Witt, I.-B. Magdău, D. J. Cole, G. Csányi, MACE-OFF23: Transferable machine learning force fields for organic molecules. arXiv:2312.15211 [physics.chem-ph] (2023). 10.1021/jacs.4c07099 PMC12123624 40387214

[38] D. M. Anstine, R. Zubatyuk, O. Isayev, AIMNet2: A neural network potential to meet your neutral, charged, organic, and elemental-organic needs. Chem. Sci. 16, 10228–10244 (2025). 10.1039/d4sc08572h PMC12057637 40342914

[39] M. Schreiner, A. Bhowmik, T. Vegge, P. B. Jørgensen, O. Winther, NeuralNEB—Neural networks can find reaction paths fast. Mach. Learn Sci. Technol. 3, 045022 (2022).

[40] E. C.-Y. Yuan, A. Kumar, X. Guan, E. D. Hermes, A. S. Rosen, J. Zádor, T. Head-Gordon, S. M. Blau, Analytical ab initio Hessian from a deep learning potential for transition state optimization. Nat. Commun. 15, 8865 (2024). 10.1038/s41467-024-52481-5 PMC11473838 39402016

[41] C. A. Grambow, L. Pattanaik, W. H. Green, Reactants, products, and transition states of elementary chemical reactions based on quantum chemistry. Sci. Data 7, 137 (2020). 10.1038/s41597-020-0460-4 PMC7210263 32385318

[42] M. Schreiner, A. Bhowmik, T. Vegge, J. Busk, O. Winther, Transition1x—A dataset for building generalizable reactive machine learning potentials. Sci. Data 9, 779 (2022). 10.1038/s41597-022-01870-w PMC9789978 36566281

[43] N. Mardirossian, M. Head-Gordon, ωB97M-V: A combinatorially optimized, range-separated hybrid, meta-GGA density functional with VV10 nonlocal correlation. J. Chem. Phys. 144, 214110 (2016). 10.1063/1.4952647 27276948

[44] Q. Zhao, S. M. Vaddadi, M. Woulfe, L. A. Ogunfowora, S. S. Garimella, O. Isayev, B. M. Savoie, Comprehensive exploration of graphically defined reaction spaces. Sci. Data 10, 145 (2023). 10.1038/s41597-023-02043-z PMC10025260 36935430

[45] E. D. Hermes, K. Sargsyan, H. N. Najm, J. Zádor, Sella, an open-source automation-friendly molecular saddle point optimizer. J. Chem. Theory Comput. 18, 6974–6988 (2022). 10.1021/acs.jctc.2c00395 36257023

[46] S. Nandi, T. Vegge, A. Bhowmik, MultiXC-QM9: Large dataset of molecular and reaction energies from multi-level quantum chemical methods. Sci. Data 10, 783 (2023). 10.1038/s41597-023-02690-2 PMC10632468 37938558

[47] E. Heid, K. P. Greenman, Y. Chung, S.-C. Li, D. E. Graff, F. H. Vermeire, H. Wu, W. H. Green, C. J. McGill, Chemprop: A machine learning package for chemical property prediction. J. Chem. Inf. Model. 64, 9–17 (2024). 10.1021/acs.jcim.3c01250 PMC10777403 38147829

[48] Q. Zhao, D. M. Anstine, O. Isayev, B. M. Savoie, Δ^2^ Machine learning for reaction property prediction. Chem. Sci. 14, 13392–13401 (2023). 10.1039/d3sc02408c PMC10686042 38033903

[49] A. L. Dewyer, A. J. Argüelles, P. M. Zimmerman, Methods for exploring reaction space in molecular systems. WIREs Comput. Mol. Sci. 8, e1354 (2018).

[50] G. Henkelman, B. P. Uberuaga, H. Jónsson, A climbing image nudged elastic band method for finding saddle points and minimum energy paths. J. Chem. Phys. 113, 9901–9904 (2000).

[51] H. Jónsson, G. Mills, K. W. Jacobsen, “Nudged elastic band method for finding minimum energy paths of transitions,” in *Classical and Quantum Dynamics in Condensed Phase Simulations* (World Scientific, 1998), p. 385–404.

[52] J. G. Brandenburg, C. Bannwarth, A. Hansen, S. Grimme, B97-3c: A revised low-cost variant of the B97-D density functional method. J. Chem. Phys. 148, 064104 (2018). 10.1063/1.5012601 29448802

[53] Y. L. A. Schmerwitz, V. Ásgeirsson, H. Jónsson, Improved initialization of optimal path calculations using sequential traversal over the image-dependent pair potential surface. J. Chem. Theory Comput. 20, 155–163 (2024). 10.1021/acs.jctc.3c01111 38154117

[54] M. Feinberg, *Foundations of Chemical Reaction Network Theory* (Springer, 2019).

[55] M. Wen, E. W. C. Spotte-Smith, S. M. Blau, M. J. McDermott, A. S. Krishnapriyan, K. A. Persson, Chemical reaction networks and opportunities for machine learning. Nat Comput. Sci. 3, 12–24 (2023). 10.1038/s43588-022-00369-z 38177958

[56] A. I. Osman, N. Mehta, A. M. Elgarahy, A. Al-Hinai, A. H. Al-Muhtaseb, D. W. Rooney, Conversion of biomass to biofuels and life cycle assessment: A review. Environ. Chem. Lett. 19, 4075–4118 (2021).

[57] M. Woulfe, B. M. Savoie, Chemical reaction networks from scratch with reaction prediction and kinetics-guided exploration. J. Chem. Theory Comput. 21, 1276–1291 (2025). 10.1021/acs.jctc.4c01401 39883589

[58] D. M. Anstine, O. Isayev, Machine learning interatomic potentials and long-range physics. J. Phys. Chem. A 127, 2417–2431 (2023). 10.1021/acs.jpca.2c06778 PMC10041642 36802360

[59] S. Yue, M. C. Muniz, M. F. Calegari Andrade, L. Zhang, R. Car, A. Z. Panagiotopoulos, When do short-range atomistic machine-learning models fall short? J. Chem. Phys. 154, 034111 (2021). 10.1063/5.0031215 33499637

[60] A. Grisafi, M. Ceriotti, Incorporating long-range physics in atomic-scale machine learning. J. Chem. Phys. 151, 204105 (2019). 10.1063/1.5128375 31779318

[61] Q. Zhao, B. M. Savoie, Simultaneously improving reaction coverage and computational cost in automated reaction prediction tasks. Nat. Comput. Sci. 1, 479–490 (2021). 10.1038/s43588-021-00101-3 38217124

[62] A. R. Todd, *Perspectives in Organic Chemistry* (Cambridge Univ. Press, 1956).

[63] C. W. Coley, W. H. Green, K. F. Jensen, Machine learning in computer-aided synthesis planning. Acc. Chem. Res. 51, 1281–1289 (2018). 10.1021/acs.accounts.8b00087 29715002

[64] S. Smidstrup, A. Pedersen, K. Stokbro, H. Jónsson, Improved initial guess for minimum energy path calculations. J. Chem. Phys. 140, 214106 (2014). 10.1063/1.4878664 24907989

[65] C. Bannwarth, S. Ehlert, S. Grimme, GFN2-xTB—An accurate and broadly parametrized self-consistent tight-binding quantum chemical method with multipole electrostatics and density-dependent dispersion contributions. J. Chem. Theory Comput. 15, 1652–1671 (2019). 10.1021/acs.jctc.8b01176 30741547

[66] A. K. Rappe, C. J. Casewit, K. S. Colwell, W. A. Goddard III, W. M. Skiff, UFF, a full periodic table force field for molecular mechanics and molecular dynamics simulations. J. Am. Chem. Soc. 114, 10024–10035 (1992).

[67] F. Neese, The ORCA program system. WIREs Comput. Mol. Sci. 2, 73–78 (2012).

[68] N. Mardirossian, M. Head-Gordon, Thirty years of density functional theory in computational chemistry: An overview and extensive assessment of 200 density functionals. Mol. Phys. 115, 2315–2372 (2017).

[69] F. Neese, An improvement of the resolution of the identity approximation for the formation of the Coulomb matrix. J. Comput. Chem. 24, 1740–1747 (2003). 10.1002/jcc.10318 12964192

[70] B. Helmich-Paris, B. de Souza, F. Neese, R. Izsák, An improved chain of spheres for exchange algorithm. J. Chem. Phys. 155, 104109 (2021). 10.1063/5.0058766 34525816

[71] A. Paszke, S. Gross, F. Massa, A. Lerer, J. Bradbury, G. Chanan, T. Killeen, Z. Lin, N. Gimelshein, L. Antiga, A. Desmaison, A. Köpf, E. Yang, Z. De Vito, M. Raison, A. Tejani, S. Chilamkurthy, B. Steiner, L. Fang, J. Bai, S. Chintala, Pytorch: An imperative style, high-performance deep learning library. arXiv:1912.01703 [cs.LG] (2019).

[72] L. Liu, H. Jiang, P. He, W. Chen, X. Liu, J. Gao, Han J, On the variance of the adaptive learning rate and beyond. arXiv:1908.03265 [cs.LG] (2019).

[73] J. Steinmetzer, S. Kupfer, S. Gräfe, pysisyphus: Exploring potential energy surfaces in ground and excited states. Int. J. Quantum Chem. 121, e26390 (2021).

[74] M. J. Frisch, G. W. Trucks, H. B. Schlegel, G. E. Scuseria, M. A. Robb, J. R. Cheeseman, G. Scalmani, V. Barone, G. A. Petersson, H. Nakatsuji, X. Li, M. Caricato, A. V. Marenich, J. Bloino, B. G. Janesko, R. Gomperts, B. Mennucci, H. P. Hratchian, J. V. Ortiz, A. F. Izmaylov, J. L. Sonnenberg, D. Williams-Young, F. Ding, F. Lipparini, F. Egidi, J. Goings, B. Peng, A. Petrone, T. Henderson, D. Ranasinghe, V. G. Zakrzewski, J. Gao, N. Rega, G. Zheng, W. Liang, M. Hada, M. Ehara, K. Toyota, R. Fukuda, J. Hasegawa, M. Ishida, T. Nakajima, Y. Honda, O. Kitao, H. Nakai, T. Vreven, K. Throssell, J. A. Montgomery Jr., J. E. Peralta, F. Ogliaro, M. J. Bearpark, J. J. Heyd, E. N. Brothers, K. N. Kudin, V. N. Staroverov, T. A. Keith, R. Kobayashi, J. Normand, K. Raghavachari, A. P. Rendell, J. C. Burant, S. S. Iyengar, J. Tomasi, M. Cossi, J. M. Millam, M. Klene, C. Adamo, R. Cammi, J. W. Ochterski, R. L. Martin, K. Morokuma, O. Farkas, J. B. Foresman, D. J. Fox, Gaussian 16 (Gaussian Inc., Wallingford CT, 2016).

[75] G. Henkelman, H. Jónsson, Improved tangent estimate in the nudged elastic band method for finding minimum energy paths and saddle points. J. Chem. Phys. 113, 9978–9985 (2000).

