# Supplementary material for: AIMNet2-rxn: A machine-learned potential for generalized reaction modeling on a millions-of-pathways scale
Source: Sci Adv. 2026 Oct 7;12(41):eaea1557. doi: 10.1126/sciadv.aea1557 (PMC13644750; doi:10.1126/sciadv.aea1557)
Supplement: Supplementary file 1 [file sciadv.aea1557_sm.pdf]

Science Advances
AAAS

Supplementary Materials for

AIMNet2-rxn: A machine-learned potential for generalized reaction modeling on a millions-of-pathways scale

Dylan M. Anstine *et al.*

Corresponding author: Olexandr Isayev, olexandr@cmu.edu

Sci. Adv. **12**, eaea1557 (2026)
DOI: 10.1126/sciadv.aea1557

This PDF file includes:

Figs. S1 to S6
Table S1
Notes S1 and S2

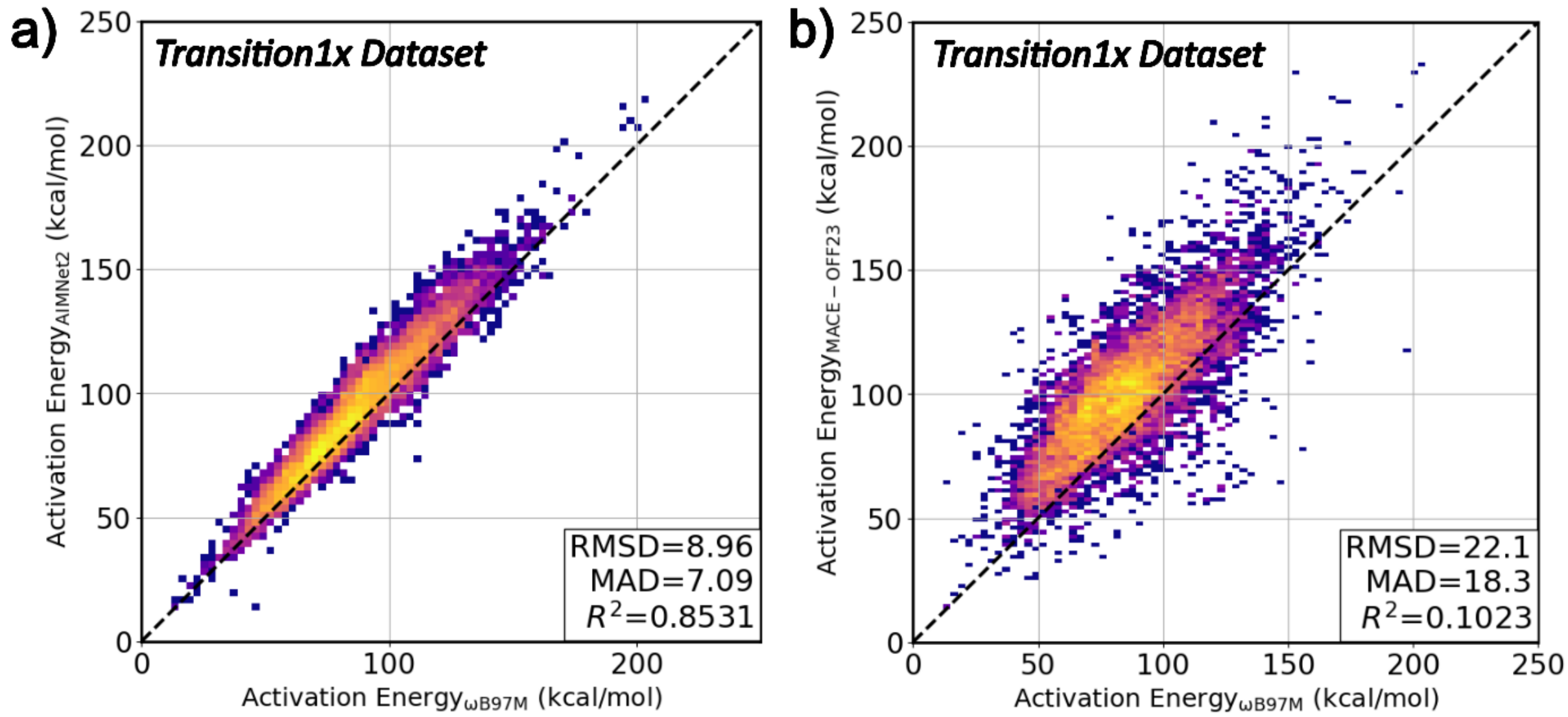

Figure S1. Illustration of the failure of existing general-purpose machine learned interatomic potentials for describing neutral, closed-shell organic reactions, which motivates the need for reactive MLIPs. The recalculated barrier heights from the Transition-1x benchmark (*42*) are shown for a) AIMNet2 (*38*) and b) MACE-OFF23 (*37*).

Table S1. Distribution of molecular system sizes comprising the AIMNet2-rxn training dataset. All systems are composed of CHNO atoms and calculated as neutral singlets.

# of atoms	# of samples	# of atoms	# of samples	# of atoms	# of samples
4	184	24	163720	44	11297
5	333	25	130851	45	10384
6	2035	26	112458	46	8398
7	18608	27	108021	47	10972
8	64851	28	111134	48	9371
9	56567	29	116457	49	5140
10	63914	30	129248	50	7424
11	113702	31	141838	51	1980
12	170259	32	172958	52	1919
13	218989	33	189325	53	4403
14	243832	34	213578	54	2440
15	287713	35	217325	55	948
16	343340	36	121952	56	2349
17	366880	37	32947	57	307
18	365845	38	27142	58	917
19	346565	39	28941	59	454
20	311223	40	23805	60	842
21	267569	41	21058	61	445
22	241432	42	15245	62	724
23	191589	43	13863		

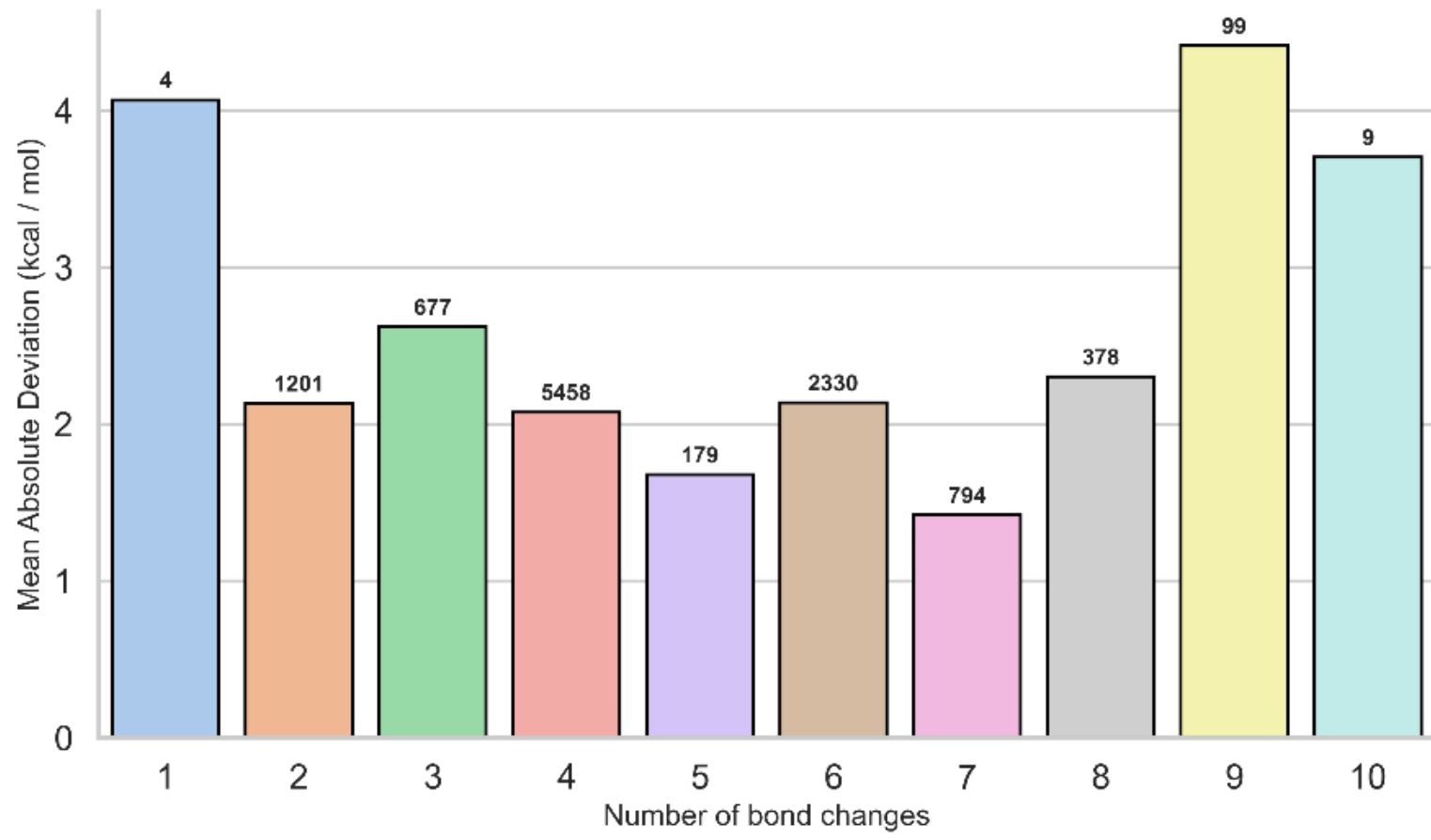

Figure S2 Accuracy of AIMNet2-rxn on the Grambow et al. (*41*) dataset delineated by the number of bond changes. Labels above each bar denote the number of samples in the benchmark set having the corresponding number of bond changes. Adjacency matrices used to calculate bond changes are inferred from the 3D geometry using RDKit, which may improperly assign ionic states for exotic valence states. Samples where either the reactant or geometry could not be converted with RDKit were removed from the analysis.

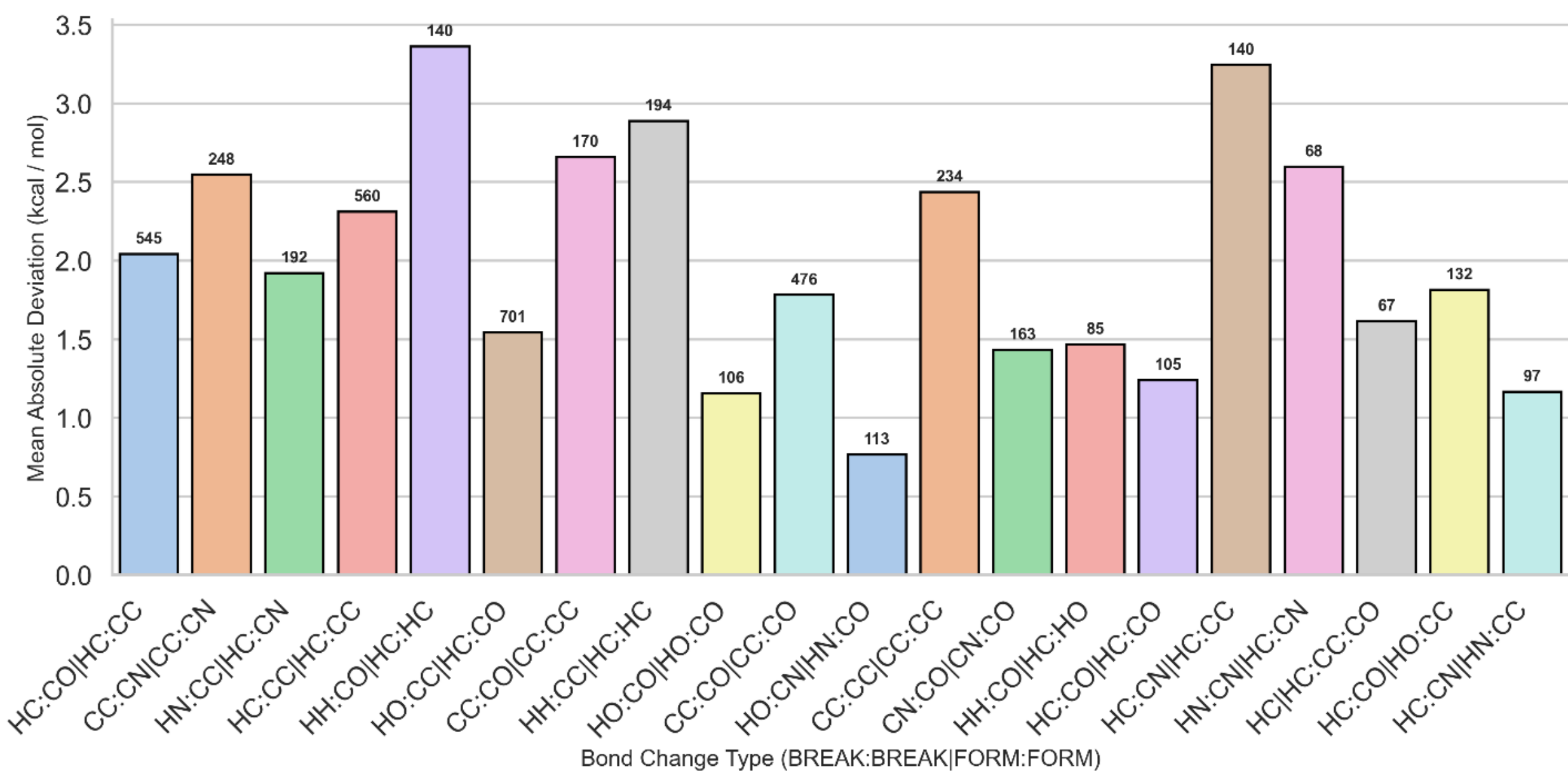

Figure S3 AIMNet2-rxn barrier height accuracy on the top 20 common bond change types from the 4-bond change superset of Grambow et al. (*41*) Element pairs before the | describe bonds being broken, where those after are bonds being formed. Labels above each bar denote the number of samples in the benchmark set belonging to the corresponding bond type. Adjacency matrices used to calculate bond changes are inferred from the 3D geometry using RDKit, which may improperly assign ionic states for exotic valence states. Samples where either the reactant or geometry could not be converted with RDKit were removed from the analysis.

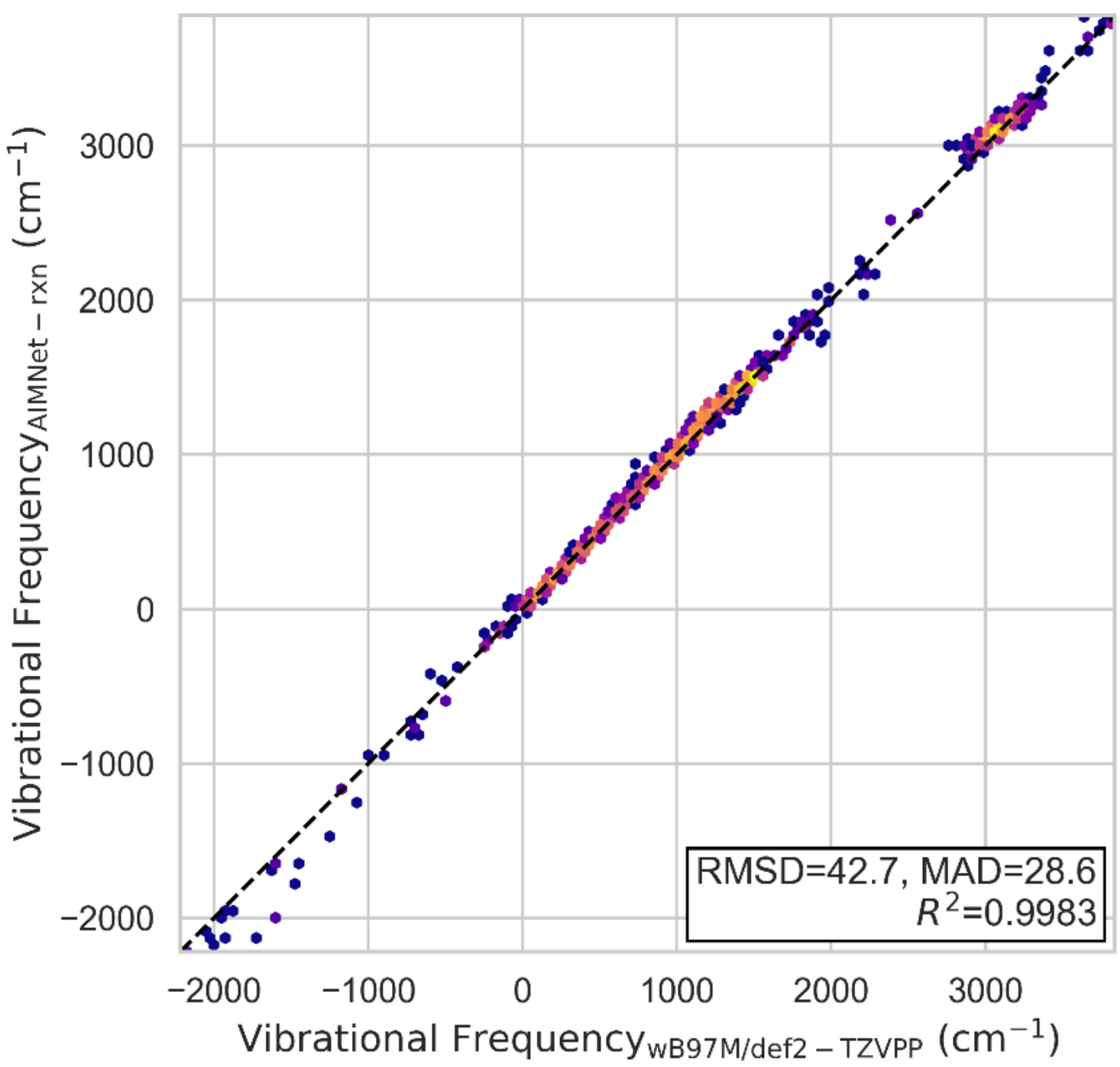

Figure S4 Parity comparison of AIMNet2-rxn predicted vibrational frequencies with respect to the density functional theory ground truth for a subset of 50 transition states from the Hermes *et al.* (*45*) dataset. Each transition state is verified to have only a single negative frequency in its spectra.

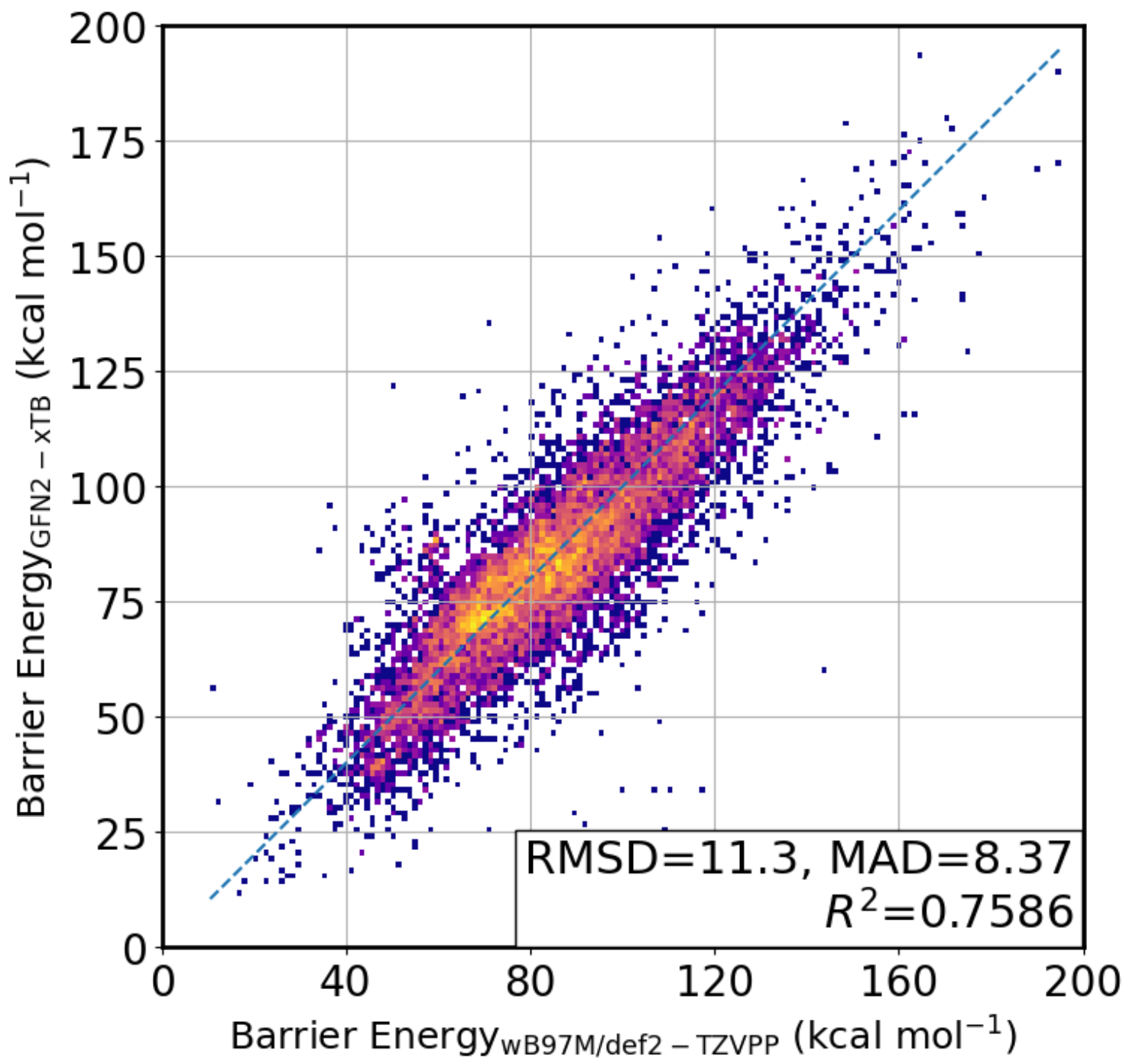

Figure S5 GFN2-xTB single-point barrier energy parity for the Grambow et al. (*41*) benchmark.

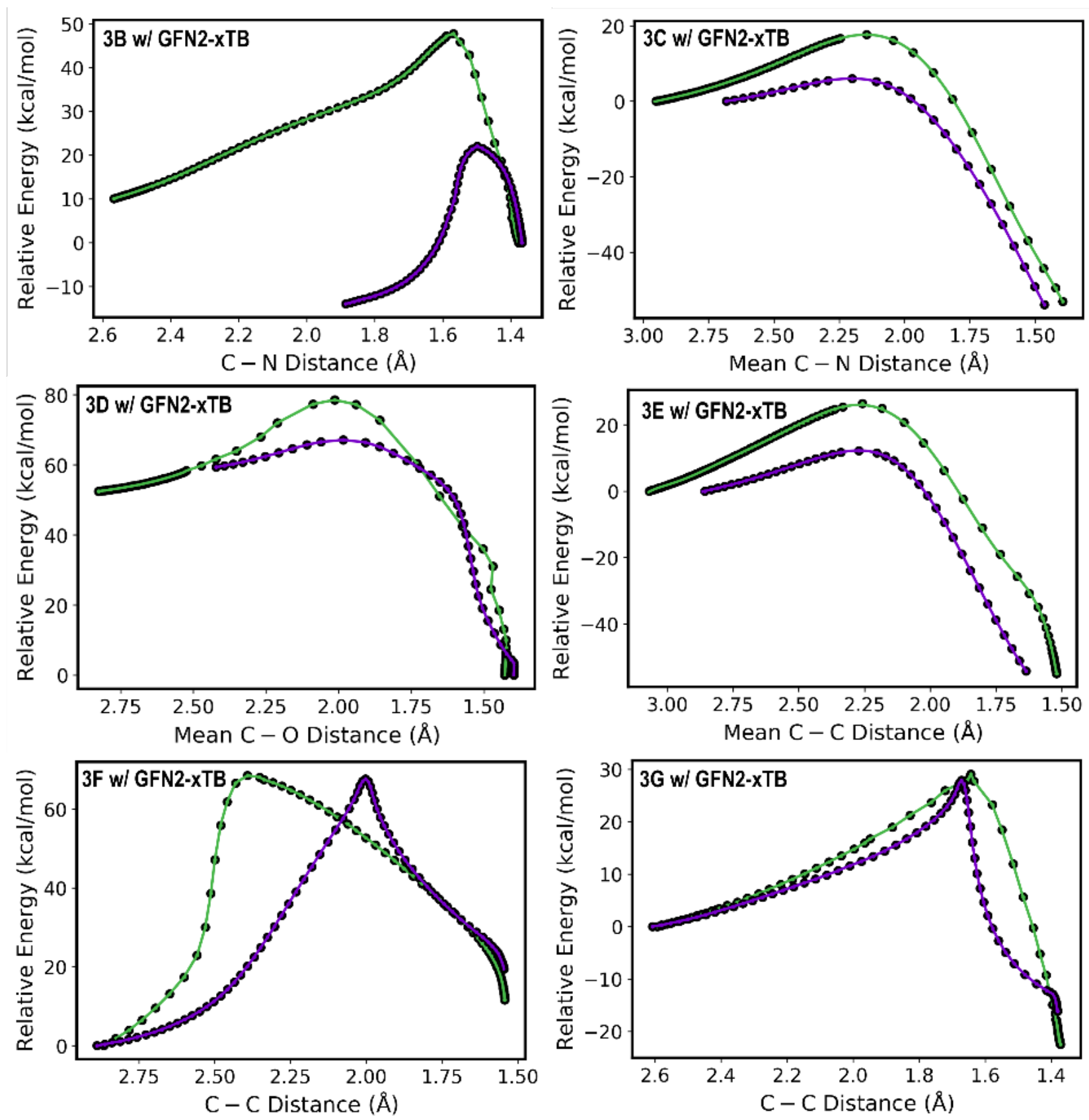

Figure S6 GFN2-xTB (purple) and ωB97M/def2-TZVPP (green) calculated IRC profiles for b) amide bond formation, c) triazole formation, d) alkene oxidation to an epoxide, e) Diels-Alder cycloaddition, f) β-keto ester formation, and g) alcohol addition to produce a carbamate.

Supplementary Note 1: Overview of Batched Nudged Elastic Band Implementation

The batch nudged elastic band (BNEB) method is designed to expand all operations used in conventional NEB (*52*) with an additional batch dimension. By formulating multiple pathway search as a single operation stacked along images and stacked along bands, the GPU-parallelized inference of AIMNet2-rxn allows for drastically accelerated reaction modeling. In this supplementary note, the algorithmic steps of BNEB are described, and a discussion of approximate benchmarking with respect to conventional DFT is described. Our method is provided as an open-source PyTorch implementation for use with current and all future versions of AIMNet2-rxn potentials.

The BNEB method encompasses a batched version of the LBFGS optimizer, batched matrix multiplication operations for rotation and alignment, batched image-dependent pair potential interpolation, and parallel evaluation of spring forces. The input to BNEB is a collection of atom-mapped reactant and product geometries. Although we require the initial conformer generation to be performed outside the BNEB framework, we provided parallel geometry optimization with LBFGS to relax the endpoints to minima on the AIMNet2-rxn potential surface before interpolation and minimum energy pathway search. Following optimization of the reactant and products, the band endpoints are linearly interpolated in their internal distance coordinate to construct the intermediate images, which are then relaxed according to a parallelized evaluation of the image dependent pair potential (IDPP) (*64*).

BNEB is conducted over an iterative loop, terminating either when all bands are converged or when the maximum number of cycles (user defined) is reached. Each loop cycle begins with translational and rotational corrections of all images in a band, which is performed to remove spurious spring forces arising from misaligned geometries in cartesian coordinates. A correction consists of shifting all systems to have a center of mass at the origin and aligning each image with the previous image in the band by multiplication with a rotation matrix obtained by singular value decomposition. It is important to note that the batched LBFGS optimizer stores a trajectory of geometry differences, gradient differences, steps, and forces that are also aligned using the same per-image rotation matrix.

Following alignment, AIMNet2-rxn energies and forces are inferred and used to determine the parallel and perpendicular forces acting on each image. During optimization of the bands, the endpoints are kept fixed, and the climbing image methodology (*51*) is initiated upon a user-defined threshold. Both energy and geometry differences are used in estimating the tangent of each image, which is outline according to the so-called “upwinding” approach as implemented in pysisyphus (*73*). The total force (parallel + perpendicular) is used for optimizing the bands in parallel with an adapted LBFGS optimizer the stores a history of shape B,H,M,N,3, where B is the batch size, H is the user defined length of history, M is the number of images, and N is the number of atoms. In our empirical testing, a history length of 7 was found to produce good convergence behavior. Bands are assessed for convergence using root-mean-squared (RMS) and maximum thresholds for the perpendicular force component. BNEB cycles are only performed on un-converged bands by applying a batch-wise mask that is updated at the end of each cycle.

Supplementary Note 2: Determination of and Considerations for BNEB vs. DFT/NEB Benchmarking

Numerous challenges exist for rigorously comparing BNEB with conventional DFT/NEB. The first point worth emphasizing is the difference between the potential energy surfaces and the algorithmic details of how energy and atomic forces are obtained. AIMNet2-rxn energy and forces are determined in a single forward and backward pass of the ML model whereas DFT requires an iterative self-consistent loop. Consequently, AIMNet2-rxn inference is relatively flat in terms of computational cost, and exhibits scaling with the number of atoms. In contrast, DFT exhibits scaling primarily in accordance with the number of electrons, and the convergence of the self-consistent loop for each image is highly dependent on the chemical system and initialization. Another point of consideration is the difference in computational resources used to perform each method: GPUs for AIMNet2-rxn and CPUs for DFT. The parallelization strategies of these hardware are fundamentally different, where multiple CPUs allow for parallelization over images in a MPI framework and GPU threads are used for massive parallelization over mathematical operations (particularly matrix multiplication). For the benchmarks reported in the main text, we used 12 images for each band with 1 CPU per image or 1 GPU for all bands in BNEB. Direct comparison between these setups, as alluded to above, is arduous, and, therefore, it is typically reduced to a measure of relative wall times, i.e., the physical time required to execute each method. The comparison of CPU and GPU resources complicates further when considering different generations of GPUs (and CPUs for that matter), which, considering the aim of our benchmark, is a consideration of secondary importance.

The largest difficulty in directly comparing BNEB with conventional NEB is the interplay between throughput and the sensitivity to each system's chemical identity. Namely, it is not feasible to evaluate (for example) ~10^6 unique systems with both NEB and BNEB. We have designed our benchmark to (1) provide some indication of the convergence behavior variable across different systems / mechanisms with DFT, but (2) focus primarily on the large difference in the scale of throughput. Thus, all DFT/NEB results are averaged over 5 systems with error bars indicating the standard deviation of wall time for each band to converge. The typical range of deviation is ~1-5 reactions per day depending on the method and system size. In contrast, we conducted BNEB efficiency tests by removing convergence criteria / masking and allotting a budget of 250 cycles, which is a cautiously prescribed budget for most cases of organic reactions. This decision was motivated by our desire to provide a reasonable lower bound for BNEB throughput while removing the variance in convergence that stems from chemical diversity. In practice, many bands tend to converge in 10s of cycles, and, therefore, applying a mask for convergence drastically increases throughput particularly if the batch size is sufficiently large to saturate the compute capabilities of the GPU being used. It should also be noted that convergence is also sensitive to the (B)NEB settings selected such as maximum step size. These can be readily tuned for single NEB tasks, but for BNEB, we attempt to select "universally good" parameters. In most cases, these are overly conservative (e.g., a maximum step size 0.02 a_o), and ongoing work focuses on the autonomous assignment of optimal settings for each band. Regardless, the summarized complexities outlined in this Supplementary Note provide justification for discussing the drastic efficiency increase afforded by BNEB on an order-of-magnitude scale instead of rigorous quantitative speedups.
